# AMPK Pathway Activation Markers in GBM: Expression Patterns and Clinical Associations in a Real-World Study

**DOI:** 10.3390/ijms27177789

**Published:** 2026-08-31

**Authors:** Federica Ferrarini, Paola Del Bianco, Martina Corrà, Tiziana Talienti, Matteo Mauceri, Daniele Boso, Giusi Romanazzi, Martina Bedeschi, Mario Caccese, Marta Padovan, Angela Guerriero, Giovanni Esposito, Isacco Desideri, Enrico Franceschi, Paola Gaviani, Michela Buglione di Monale e Bastia, Gian Luca De Salvo, Alba Fiorentino, Anna Tesei, Tommaso Mazza, Giuseppe Lombardi, Stefano Indraccolo

**Affiliations:** 1 Basic and Translational Oncology Unit, Veneto Institute of Oncology IOV-IRCCS, 35128 Padua, Italy; federica.ferrarini@iov.veneto.it (F.F.);; 2 Clinical Research Unit, Veneto Institute of Oncology IOV-IRCCS, 35128 Padua, Italy; 3 Medical Oncology 1, Veneto Institute of Oncology IOV-IRCCS, 35128 Padua, Italy; 4 UOC Radioterapia Oncologica, Ente Ecclesiastico Ospedale F Miulli, 70021 Acquaviva delle Fonti (BA), Italy; 5 Biosciences Laboratory, Istituto Scientifico Romagnolo per lo Studio e la Cura dei Tumori (IRST) IRCCS, 47014 Meldola (FC), Italy; 6 Surgical Pathology and Cytopathology Unit, Department of Medicine-DIMED, University of Padua School of Medicine, 35128 Padua, Italy; 7 Immunology and Molecular Oncology Diagnostics, Veneto Institute of Oncology IOV-IRCCS, 35128 Padua, Italy; 8 Radiation Oncology, Breast Unit, Azienda Ospedaliero-Universitaria Careggi, 50134 Florence, Italy; 9 Department of Experimental and Clinical Biomedical Sciences “M. Serio”, University of Florence, 50134 Florence, Italy; 10 Nervous System Medical Oncology Department, IRCCS Istituto delle Scienze Neurologiche di Bologna, 40139 Bologna, Italy; 11 Neuroncology Unit, Fondazione IRCCS Istituto Neurologico Carlo Besta, 20133 Milan, Italy; 12 Department of Radiation Oncology, Università di Brescia and Spedali Civili Hospital, 25123 Brescia, Italy; 13 Dipartimento di Medicina e Chirurgia, Università LUM, 70010 Casamassima (BA), Italy; 14 Laboratory of Bioinformatics, Fondazione IRCCS Casa Sollievo della Sofferenza, 71013 San Giovanni Rotondo (FG), Italy; 15 Department of Surgery, Oncology and Gastroenterology, University of Padua, 35128 Padua, Italy

**Keywords:** glioblastoma, regorafenib, pACC, immunohistochemistry, digital pathology

## Abstract

Phosphorylated ACC (pACC), a downstream effector of the LKB1/AMPK pathway, may support tumor bioenergetics and exert a tumor-promoting role in glioblastoma (GBM). In the randomized REGOMA trial, pACC expression was associated with improved overall survival (OS) in GBM patients treated with regorafenib, but its validity in large cohorts is unclear. This study aimed to characterize pACC and phosphorylated AMPK (pAMPK) expression in a real-world GBM cohort and to assess their prognostic and predictive value in patients receiving second-line regorafenib or fotemustine/lomustine. In this retrospective, multicenter translational study, pACC and pAMPK were assessed by immunohistochemistry on FFPE tumor sections from 174 IDH-wild-type GBM patients treated with regorafenib (n = 84) or fotemustine/lomustine (n = 90). Staining was performed with a validated anti-pACC monoclonal antibody and quantified by digital pathology. Survival analyses included univariable and multivariable Cox proportional hazards models, with interaction testing. pACC and pAMPK were expressed in 85.6% and 95.4% of samples, respectively, with heterogeneous spatial patterns. Neither marker was significantly associated with OS in the univariable analyses, and neither retained independent prognostic value in multivariable analysis. Regorafenib was associated with significantly longer OS than alkylating agents (10.4 vs. 6.3 months; *p* = 0.0021). However, the treatment-by-pACC interaction test was not statistically significant (adjusted *p* = 0.610), providing no formal evidence of a differential treatment effect by pACC status. Although pACC-positive expression was enriched among patients experiencing longer overall survival under regorafenib, formal treatment-by-biomarker interaction was non-significant. These exploratory findings indicate that pACC is not an established predictive biomarker and warrant prospectively powered evaluation.

## 1. Introduction

Regorafenib is a small-molecule multi-kinase inhibitor that targets vascular endothelial growth factor receptors (VEGFR-1–3), angiopoietin-1 receptor (TIE2), platelet-derived growth factor receptor-β (PDGFR-β), fibroblast growth factor receptors (FGFR), and oncogenic kinases such as KIT, RET, RAF1, and BRAF, thereby counteracting tumor, vascular, and stromal signaling pathways [1,2]. Based on its broad kinase-inhibitory profile and encouraging preclinical activity in glioblastoma, *IDH* (isocitrate dehydrogenase)-wild-type (GBM) models, regorafenib has been evaluated in the recurrent GBM setting [3,4]. The randomized, multicenter phase II REGOMA trial first demonstrated that regorafenib significantly improves overall survival (OS) compared with lomustine in patients with relapsed *IDH*-wild-type GBM, establishing this drug as a viable second-line option in this population [5].

Building on these clinical findings, the REGOMA translational program identified several in situ biomarkers related to tumor metabolism and the liver kinase B1 (LKB1)/AMP-activated protein kinase (AMPK) pathway that might modulate response to antiangiogenic therapy. In particular, the activation status of AMPK can be interrogated by immunohistochemical assessment of phosphorylated ACC at Ser79 (pACC), a canonical downstream target whose phosphorylation reflects AMPK-mediated inhibition of fatty acid synthesis and metabolic adaptation to energy stress [6]. In our recent study, analysis of tumor sections from the REGOMA cohort showed that pACC expression was associated with a clinically meaningful benefit in terms of OS among patients treated with regorafenib, whereas no significant association was observed in the lomustine-treated arm, suggesting that basal activation of the AMPK/pACC axis may identify a subset of GBM patients more likely to respond to this drug [6]. More recent clinical studies have further reinforced the relevance of regorafenib in recurrent GBM. The large, multicenter REGOMA-OSS observational study confirmed that regorafenib yields a median OS and toxicity profile comparable to those observed in the original REGOMA trial, thus supporting its activity in routine clinical practice [7]. At the same time, experimental work has highlighted the complex, context-dependent role of AMPK in GBM, showing that chronic oncogenic stress can drive sustained AMPK activation in brain tumors, which in turn reprograms tumor bioenergetics via HIF1α and GABPA-dependent transcriptional programs and sustains tumor growth [8]. These findings highlight AMPK activation as a tumor-promoting pathway in the context of brain tumors and suggest that AMPK inhibitors deserve consideration as novel therapeutics in this clinical setting. However, in previous studies, the pattern of expression of AMPK markers in GBM has been characterized only in small cohorts of patients, and its prognostic value is not firmly established. Moreover, despite the encouraging data from the REGOMA trial, the robustness and generalizability of pACC as a predictive biomarker for regorafenib response in recurrent GBM need to be further validated in independent, real-world cohorts. Here, we report a retrospective, multicenter real-world study designed to chart patterns of expression of AMPK markers in GBM and to further investigate the prognostic and predictive value of pACC expression in tumor tissue with respect to clinical outcomes in patients with relapsed GBM treated with regorafenib.

## 2. Results

### 2.1. Refinement of pACC IHC Staining

As a prerequisite for cross-cohort validation of the REGOMA-based pACC readout, we harmonized the immunostaining and scoring pipeline in the present series. Primary antibody incubation was prolonged to 1 h (from 15 min in the published REGOMA protocol [6]), the digital scoring algorithm was refined by adjusting threshold settings, and a head-to-head comparison of polyclonal and monoclonal anti-pACC antibodies was performed (Figure S1). The monoclonal antibody was considered to enhance analytical performance, in view of its potentially higher target selectivity and reduced inter-lot variability, which are important considerations for biomarker development.

Antibody specificity was tested by selectively silencing the two transcripts (*ACACA* and *ACACB*) encoding ACC isoforms in LN229 glioblastoma cells and assessing changes in the pACC signal by Western blot with either the monoclonal or polyclonal reagent. Upon *ACACA* silencing, the pACC band detected with the mAb was markedly reduced, whereas *ACACB* silencing produced only a minor decrease, indicating preferential recognition of isoform 1 encoded by *ACACA*; in contrast, the polyclonal antibody showed a broader isoform pattern, consistent with its reported reactivity against both *ACACA-* and *ACACB*-encoded proteins (Figure S1A). Since ACACA is considered the predominant ACC isoform expressed in GBM cells [9,10], we selected the more isoform-selective mAb to minimize potential cross-reactivity.

Immunostainings with monoclonal and polyclonal antibodies were performed in parallel on 57 tumor samples and quantified as in the REGOMA study, using the sum of the percentages of tumor cells with 2+ and 3+ staining intensity (Figure S1B). The two antibodies yielded strongly concordant scores (Spearman’s rho 0.802, 95% CI 0.680–0.881, *p* < 0.0001). Bland–Altman analysis, computed as monoclonal minus polyclonal, showed a small mean difference (bias 2.32) with a standard deviation of 17.2 and 95% limits of agreement from −31.3 to 36.0 (Figure S1D), indicating highly consistent ranking but differences of up to approximately ±30–35 percentage points in individual cases. Notably, the largest discordances were observed in samples with intermediate positivity scores (approximately 20–50%), where inter-antibody variability was most pronounced (Figure S1C). To complement the Bland–Altman analysis, we assessed agreement between the monoclonal and polyclonal anti-pACC antibodies at the applied 5% positivity threshold (Figure S1B). Categorical agreement was substantial (89.5%, 51/57; Cohen’s κ = 0.75, 95% CI 0.56–0.94), with six samples (10.5%) reclassified across the threshold depending on the antibody used (five monoclonal-positive/polyclonal-negative; one monoclonal-negative/polyclonal-positive), consistent with the sample-level differences observed in the Bland–Altman analysis. These discordances most likely reflect subtle differences in epitope recognition between the two antibodies for samples close to the positivity cut-off. We further evaluated inter-run variability of pACC immunostaining. Three representative samples spanning the range of pACC positivity were re-stained with both antibodies on three separate occasions (1, 5 and 7 days after slide sectioning) and quantified by the same observer using the same algorithm (Figure S1C); two of these samples (1763-6 and 1763-7) belonged to the present validation series, while the third (142-80) was drawn from the earlier REGOMA cohort [6], allowing the reproducibility assessment to span both sample sets. Reproducibility was good for samples with unequivocally high or low positivity (coefficient of variation generally <30%), while greater inter-run variability (CV up to approximately 100%) was observed for samples/time-points in the low-to-intermediate positivity range, in line with the higher inter-antibody discordance observed near the 5% threshold. No formal adjudication or re-reading protocol was applied to intermediate or discordant cases; these were retained as originally scored, and their higher variability is discussed as an inherent limitation of the assay in this range.

On the basis of these data, all subsequent analyses were performed with the anti-pACC mAb under the optimized conditions. Although both antibodies showed overall acceptable agreement, the monoclonal antibody was further preferred in view of its potential translatability to clinical practice, where the inherently lower inter-lot variability of monoclonal reagents represents a critical advantage for assay standardization and reproducibility across laboratories.

### 2.2. Patient Characteristics

Archived tumor specimens were collected from the REGOMA-OSS cohort [7] and from additional institutional series of patients with recurrent GBM receiving regorafenib or lomustine or fotemustine as second-line therapy. A total of 211 FFPE samples were available; in line with current WHO recommendations, *IDH*-mutant cases (n = 6 in the regorafenib arm and n = 1 in the lomustine/fotemustine arm) and *IDH*-unknown cases (n = 16 in the regorafenib arm and n = 14 in the lomustine/fotemustine arm) were excluded, yielding 90 lomustine/fotemustine-treated and 84 regorafenib-treated tumors for analysis. Baseline characteristics were broadly similar across treatment groups and are shown in Table 1. Within the control arm, demographic, clinical, and molecular characteristics were comparable between fotemustine (n = 66) and lomustine (n = 24), except for a higher proportion of ECOG PS 0–1 in the fotemustine subgroup (93.9% vs. 68.2%; *p* = 0.008, Table S1). Given their shared mechanism as alkylating nitrosoureas and overall baseline similarity, fotemustine and lomustine were considered as a single pooled control cohort (N = 90). Compared with the combined control group, regorafenib-treated patients had a slightly lower median age (60.6 vs. 63.5 years; *p* = 0.112) and a lower proportion of males (56.0% vs. 70.0%; *p* = 0.055), though neither difference was statistically significant. Notably, the regorafenib cohort presented with a significantly better baseline ECOG performance status (100% vs. 85.9% with PS 0–1; *p* < 0.001), and a lower median expression of pACC (26.2 vs. 46.5; *p* = 0.009) and pAMPK (53.0 vs. 71.2; *p* = 0.004). *MGMT* promoter methylation rates at diagnosis were broadly similar (50.0% vs. 56.1%; *p* = 0.446).

Among surviving patients, the overall median follow-up was 16.7 months (Q1, Q3: 12.1, 30.6). Stratified by second-line regimen, median follow-up among surviving patients was 16.7 months (Q1, Q3: 11.3, 32.8) for Regorafenib (n = 8) and 19.8 months (Q1, Q3: 16.7, 22.8) for Fotemustine (n = 2). All patients receiving Lomustine had reached the primary survival endpoint (n = 24/24 deaths).

### 2.3. Patterns of Biomarker Expression in GBM Samples and Correlation Analysis

Digital pathology data for all biomarkers and tumor samples analyzed are available in the Zenodo repository at https://doi.org/10.5281/zenodo.20282645. We performed IHC of pACC, a direct downstream target of AMPK, to investigate its pattern of expression and prognostic/predictive value in a real-world cohort of GBM samples (Figure S2). Expression of pAMPK, a marker associated with the LKB1/AMPK pathway activation, was also investigated in the same tumor samples. Finally, in a subset of samples (n = 28 in the regorafenib arm and n = 31 in the fotemustine/lomustine arm) we studied the expression of ACC and investigated its correlation with pACC. IHC positivity for pACC and pAMPK was defined as the sum of the proportions of cells scoring 2+ and 3+ intensity, consistent with the approach adopted in our previous work [6]. Quantification of pACC expression in representative samples by the digital pathology workflow used in this study is shown in Figure S3.

A cut-off of 5% was applied to classify tumors as positive or negative for both markers. In the fotemustine/lomustine group (n = 90), nine samples (9/90, 10.0%) were classified as pACC-negative (pACC ≤ 5%), while no pAMPK-negative (pAMPK ≤ 5%) cases were observed. In the regorafenib group (n = 84), 16 cases (16/84, 19.0%) were pACC-negative and eight (8/84, 9.5%) were pAMPK-negative. Among pACC-positive (pACC > 5%) cases, the distribution of positivity scores differed between treatment arms. In the fotemustine/lomustine group, pACC positivity ranged from 6.24% to 98.11%, with a median of 50.66% (Q1–Q3: 28.68–71.55%). In the regorafenib group, pACC positivity ranged from 6.19% to 91.06%, with a median of 41.82% (Q1–Q3: 22.94–60.30%). In the fotemustine/lomustine group, pAMPK positivity ranged from 10.67% to 99.82%, with a median of 71.17% (Q1–Q3: 50.46–86.85%). Among regorafenib-treated cases considered as pAMPK-positive (pAMPK > 5%), pAMPK positivity ranged from 7.52% to 97.30%, with a median of 57.05% (Q1–Q3: 37.93–80.97%). To assess whether block age (year of surgery, 2007–2025) influenced marker positivity, we performed a Cochran–Armitage trend test across the full range of surgery years for both pACC and pAMPK. No significant trend in positivity rate was observed as a function of block age for either marker (pACC: z = 0.78, *p* = 0.438; pAMPK: z = 1.56, *p* = 0.119; Supplementary Table S2), arguing against a substantial confounding effect of block storage duration on phosphoprotein immunoreactivity in this cohort.

In general, pAMPK and pACC expression was detected in the cytoplasm of tumor cells, as expected. Heterogeneous patterns of pACC and pAMPK expression were observed (Figure S4A–C). In the fotemustine/lomustine group, pACC-positive samples displayed a peri-necrotic pattern in 13/81 (16%) cases, a peri-vascular pattern in 39/81 (48.2%), a homogeneous pattern in 11/81 (13.6%), and a prevalent pattern not assignable to any of the above categories (“mixed”) in 18/81 (22.2%). These cases showed a recognizable spatial distribution of positive cells, but without a clear predominance around vessels, necrotic areas, or throughout the tumor, and therefore could not be confidently assigned to a single topographical category. pAMPK-positive samples in the same group showed a peri-necrotic pattern in 11/90 (12.2%), a peri-vascular pattern in 21/90 (23.3%), a homogeneous pattern in 32/90 (35.6%), and a mixed pattern in 26/90 (28.9%). In the regorafenib group, pACC-positive samples displayed a peri-necrotic pattern in 6/68 (8.9%), a peri-vascular pattern in 26/68 (38.2%), a homogeneous pattern in 10/68 (14.7%), and a mixed pattern in 26/68 (38.2%; Figure S4A–C). pAMPK-positive samples showed a peri-necrotic pattern in 5/76 (6.6%), a peri-vascular pattern in 19/76 (25.0%), a homogeneous pattern in 20/76 (26.3%), and a mixed pattern in 32/76 (42.1%). Spatial expression patterns of pACC and pAMPK were concordant in 39/81 (48.2%) positive samples in the fotemustine/lomustine group and in 52/67 (77.6%) positive samples in the regorafenib group, while discordant patterns were observed in 42/81 (51.9%) and 15/67 (22.4%) positive cases, respectively. The peri-necrotic distribution was consistent with the canonical role of the LKB1/AMPK pathway as a sensor of ATP levels [11], while the preferential localization of pACC- and pAMPK-positive cells in highly vascularized regions of the tumor section could suggest a potential association between AMPK pathway activation and the peri-vascular microenvironment. In addition, the homogeneous pattern suggests that in a subset of cases pACC and pAMPK expression may be coupled to oncogenic stress, as hypothesized in previous studies [12,13].

To investigate the relationship between pAMPK, pACC, and ACC, we performed Spearman rank correlation analyses across the patient samples (Figure S5). Analysis of the unstratified cohort revealed a moderate, significant positive correlation between pAMPK and pACC expression (Spearman’s rho = 0.47, 95% CI: 0.34, 0.58, *p* < 0.001) (Figure S5A). When stratifying the cohort by treatment regimen, the positive association between pAMPK and pACC remained robust and statistically significant in both groups (Figure S5C,D). In contrast, a weaker but statistically significant positive correlation was observed between ACC and pACC levels across all samples (Spearman’s rho = 0.31, 95% CI: 0.03, 0.54, *p* = 0.028) (Figure S5B). When stratified by treatment regimen, this positive trend between ACC and pACC persisted but did not reach statistical significance in either the fotemustine/lomustine arm (Spearman’s rho = 0.38, 95% CI: −0.04, 0.69, *p* = 0.068) or the regorafenib arm (Spearman’s rho = 0.31, 95% CI: -0.09, 0.62, *p* = 0.120).

### 2.4. Assessment of the Prognostic and Predictive Value of pACC and pAMPK

Second-line treatment with regorafenib was associated with a statistically significant OS benefit compared to the pooled fotemustine/lomustine control group (Table 2). Patients treated with regorafenib achieved a median OS of 10.4 months (95% CI: 8.6, 13.3) compared to 6.3 months (95% CI: 5.4, 7.7) for those receiving fotemustine/lomustine. In univariable Cox regression, regorafenib significantly reduced the risk of death by 38% (HR = 0.62, 95% CI: 0.45, 0.84; *p* = 0.0023). This survival advantage remained statistically significant after multivariable adjustment, conferring a 31% reduction in mortality risk (adjusted HR = 0.69, 95% CI: 0.49, 0.97; *p* = 0.0344).

Conversely, baseline expression levels of pACC and pAMPK at the standard 5.0% threshold were not significantly associated with OS, in line with previous observations in the REGOMA trial [6]. Patients with high pACC expression (>5.0%) had a median OS of 8.3 months (95% CI: 7.3, 9.4) compared to 7.5 months (95% CI: 5.0, 12.2) in those with low expression (≤5.0%). No significant association with OS was observed in either univariable (HR = 0.86, 95% CI: 0.56, 1.33; *p* = 0.4970) or multivariable analysis (adjusted HR = 1.02, 95% CI: 0.60, 1.72; *p* = 0.9516). Patients classified as positive for pAMPK expression (>5.0%) demonstrated a median OS of 8.1 months (95% CI: 7.0, 9.1) versus 7.0 months (95% CI: 2.6, 14.5) for pAMPK-negative (≤5.0%). Expression levels showed no prognostic significance in univariable (HR = 0.79, 95% CI: 0.37, 1.69; *p* = 0.5390) or multivariable models (adjusted HR = 0.73, 95% CI: 0.30, 1.76; *p* = 0.4776).

To evaluate whether the biomarker prognostic value was sensitive to threshold selection, a sensitivity analysis was performed using median expression values as alternative cut-offs. Categorizing expression levels by median values reinforced the lack of prognostic value for both biomarkers: pACC expression showed no significant association with OS in either univariable (HR = 0.86, 95% CI: 0.63, 1.17; *p* = 0.3320) or multivariable models (adjusted HR = 0.90, 95% CI: 0.64, 1.27; *p* = 0.5382). Similarly, the pAMPK median split failed to demonstrate prognostic relevance in univariable (HR = 0.93, 95% CI: 0.69, 1.27; *p* = 0.1030) and multivariable analyses (adjusted HR = 0.89, 95% CI: 0.63, 1.25; *p* = 0.4959). Importantly, the overall survival benefit associated with regorafenib remained robust and statistically significant when adjusting for median-dichotomized biomarker levels (adjusted HR = 0.68, 95% CI: 0.48, 0.96; *p* = 0.0277).

To confirm that the observed overall survival benefit of regorafenib was not driven by baseline prognostic imbalances—specifically, the exclusive presence of patients with poorer performance status (ECOG PS 2–3) in the fotemustine/lomustine arm—we performed rigorous sensitivity analyses. By restricting the cohort to patients with ECOG PS 0–1 to guarantee strict common support and applying Multiple Imputation by Chained Equations (MICE) coupled with Inverse Probability of Treatment Weighting (IPTW), we achieved excellent covariate balance. Within this highly controlled framework, regorafenib remained a statistically significant independent predictor of improved OS (Pooled IPTW-adjusted HR = 0.64; 95% CI: 0.44, 0.93; *p* = 0.021). This survival benefit was fully corroborated by a pooled multivariable Cox regression model on the same restricted cohort (Adjusted HR = 0.54; 95% CI: 0.37, 0.79; *p* = 0.002). Comprehensive methodological details and causal diagnostics are provided in the Supplementary Results paragraph, the Supplementary Materials and Methods, Supplementary Table S3, Supplementary Figures S6 and S7.

In the progression-free survival analysis, no statistically significant difference was observed between treatment arms (Table S4). Patients in the regorafenib group had a median PFS of 2.6 months (95% CI: 2.2, 3.7) compared to 2.4 months (95% CI: 2.3, 2.7) in the pooled fotemustine/lomustine control group. Treatment with regorafenib was not associated with a significant reduction in the risk of progression in univariable Cox analysis (HR = 0.85, 95% CI: 0.63, 1.15; *p* = 0.3010) or after multivariable adjustment (adjusted HR = 0.91, 95% CI: 0.66, 1.27; *p* = 0.5880). Similarly, neither biomarker was significantly associated with PFS. Median PFS was 2.4 months (95% CI: 2.3, 3.0) for patients with high pACC expression (>5.0%) versus 2.6 months (95% CI: 2.3, 3.2) for those with low expression (≤5.0%). No significant association was found in univariable (HR = 1.22, 95% CI: 0.79, 1.88; *p* = 0.3770) or multivariable analysis (adjusted HR = 1.17, 95% CI: 0.68, 2.00; *p* = 0.5633). Patients with high pAMPK expression (>5.0%) had a median PFS of 2.4 months (95% CI: 2.3, 2.8) compared to 5.0 months (95% CI: 1.2, 12.2) for those with low expression (≤5.0%). Expression levels showed no prognostic significance in univariable (HR = 1.51, 95% CI: 0.71, 3.22; *p* = 0.2890) or multivariable models (adjusted HR = 1.38, 95% CI: 0.54, 3.49; *p* = 0.4957).

Interaction analyses were conducted to evaluate whether the efficacy of second-line treatments on OS and PFS was influenced by the expression levels of pACC or pAMPK.

**Figure 1:**
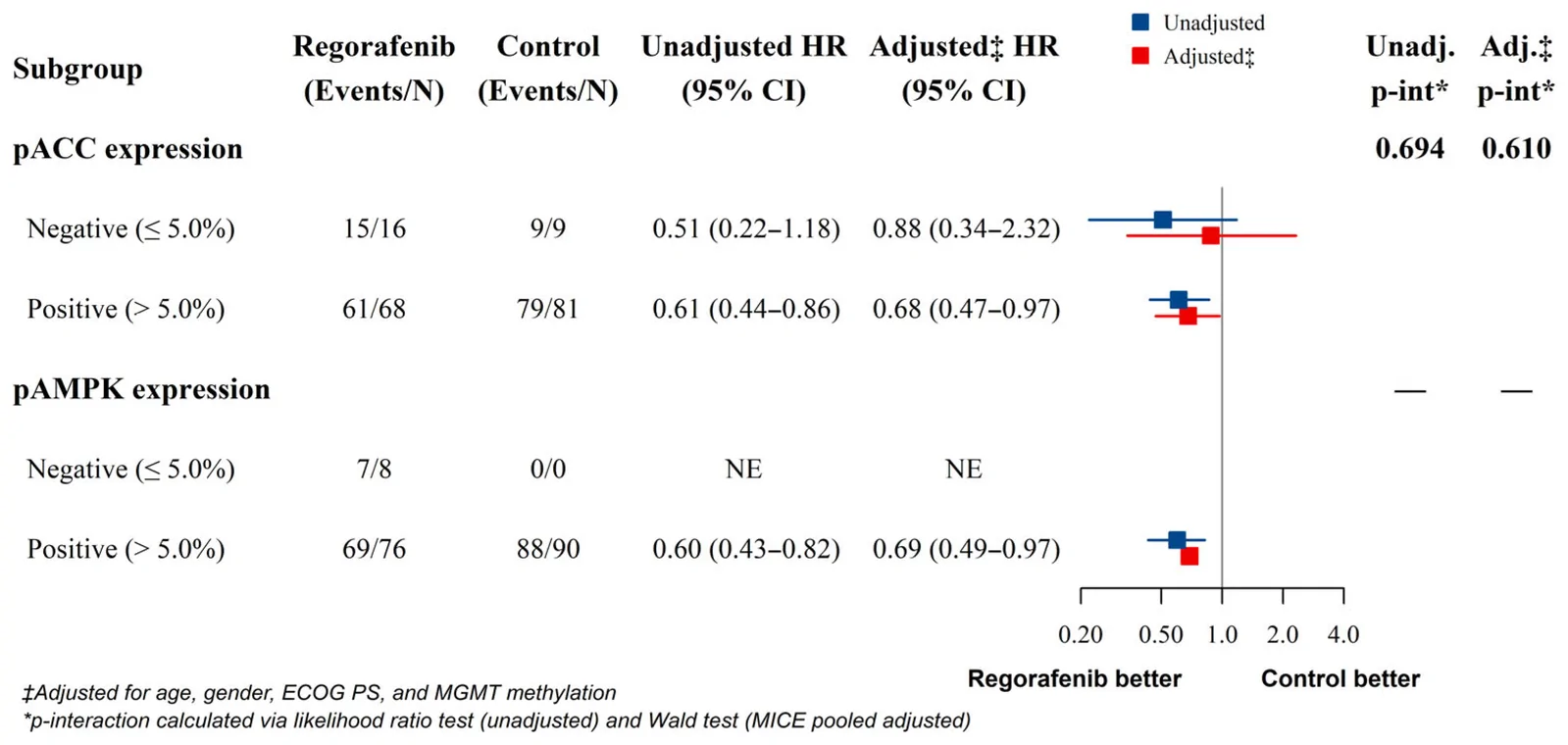
Subgroup analysis of overall survival according to baseline pACC and pAMPK expression levels. Forest plot displaying unadjusted and adjusted hazard ratios (HR) for overall survival (OS) comparing second-line regorafenib with pooled control treatment (fotemustine/lomustine). For each subgroup, the number of events per total patients (Events/N), unadjusted HR (95% CI), multivariable-adjusted HR (95% CI), and interaction *p*-values are reported. CI, confidence interval; HR, hazard ratio; NE, not estimable. HRs < 1.0 indicate a survival benefit favoring regorafenib.

Regorafenib consistently conferred an OS advantage over fotemustine/lomustine across both pACC expression strata (≤5.0% vs. >5.0%). Formal testing revealed no statistically significant interaction between treatment strategy and pACC expression in univariable analysis (*p*-value for interaction = 0.694). Similarly, in the multivariable pooled Cox regression model adjusting for baseline confounders, the interaction term remained non-significant (*p*-value for interaction = 0.610). These findings confirm that the survival benefit of regorafenib is not significantly modulated by baseline pACC expression (Figure 1).

The complete absence of fotemustine/lomustine patients in the pAMPK-negative subgroup limits the ability to draw definitive conclusions about that specific biomarker’s predictive role.

Stratification by pACC status showed a numerical trend toward lower hazard ratios for PFS in the ≤5.0% stratum (unadjusted HR = 0.71, 95% CI: 0.31, 1.63; adjusted HR = 0.71, 95% CI: 0.29, 1.77) compared to the >5.0% stratum (unadjusted HR = 0.90, 95% CI: 0.65, 1.24; adjusted HR = 0.92, 95% CI: 0.66, 1.30). However, tests for interaction confirmed no significant effect modification by pACC status on PFS in either unadjusted (*p*-value for interaction = 0.605) or multivariable-adjusted analyses (*p*-value for interaction = 0.587). For pAMPK status, PFS could not be evaluated in the ≤5.0% subgroup due to zero patients/events in the control arm (Figure S8).

## 3. Discussion

Previous research has highlighted the critical biological role of the AMPK pathway in glioma. Independent reports have concluded that sustained AMPK activation is a hallmark of GBM, facilitating the high metabolic demands of malignant cells and linking this pathway to the aggressive oncogenic signaling characteristic of these tumors [8,13]. Mechanistically, AMPK enhances *HIF1α* and *GABPA* transcription to support GBM bioenergetics. However, these studies lacked a broad investigation of AMPK expression markers in large patient cohorts, as well as a definitive assessment of the pathway’s prognostic and predictive value. Our study addresses this knowledge gap by analyzing pAMPK and pACC expression in 174 IDH-wild-type GBM samples.

Our findings reveal that pACC and pAMPK expression levels are moderately positively correlated (Spearman’s rho = 0.47)—consistent with pAMPK reflecting LKB1-mediated AMPK activation and pACC serving as a downstream marker of AMPK activity—and exhibit remarkably heterogeneous patterns. Notably, a small subset of tumors (9.1% and 10.9%, respectively) displayed reinforced pAMPK and pACC expression in viable cells surrounding necrotic areas.

This observation aligns with the canonical “Go-or-Grow” hypothesis [14]: glioblastoma cells must constantly balance proliferation and migration based on nutrient availability. Mechanistically, under normoglycemic conditions, high levels of miR-451 suppress the LKB1/AMPK pathway, allowing mTOR to drive rapid cell division [15].

Conversely, under glucose deprivation—typically in areas where the tumor outpaces its blood supply—miR-451 levels drop, thus releasing the LKB1/AMPK pathway. Once activated, this axis halts energy-intensive division and triggers invasion into the surrounding parenchyma to seek better nutrient sources. Prior functional studies in glioma cell models have identified LKB1/AMPK pathway activation under glucose deprivation as a driver of the invasive phenotype [15]. The peri-necrotic pACC/pAMPK expression pattern observed in a subset of our samples is consistent with this mechanism and may reflect pathway activation contributing to an invasive phenotype under metabolic stress. However, the present study is based on static immunohistochemical assessment and did not directly evaluate invasion, migration, or causal AMPK dependence in this cohort, and results should be considered hypothesis-generating. Notably, a substantial proportion of our GBM samples exhibited either a homogeneous expression pattern or a heterogeneous pattern with reinforced pACC/pAMPK expression in viable fields surrounding blood vessels. These patterns predominated in our cohort (49.3% and 52.7%, for pACC and pAMPK, respectively), supporting the hypothesis that AMPK activation is essential for maintaining cell viability and growth under the oncogenic stress inherent to GBM [8,13].

Altogether, the vast majority of tumor samples expressed these AMPK activation markers (85.6% and 95.4% for pACC and pAMPK, respectively). However, IHC positivity, pathway phosphorylation status and drug sensitivity are not equivalent. Therefore, this widespread expression should therefore be regarded as a rationale for further functional studies investigating AMPK pathway dependency, rather than as evidence supporting broad therapeutic applicability of AMPK-targeting strategies. Finally, this interpretation should be considered in light of the substantial increase in immunostaining sensitivity introduced by the revised assay, which used a different monoclonal antibody and a prolonged incubation time. This methodological change likely reduced the number of pACC-negative cases in the control arm to only nine patients, and therefore represents an important limitation when interpreting subgroup distribution. Preanalytical variables upstream of block preparation were not centrally recorded, and staining was performed across multiple batches; although sections were uniformly prepared and stained in a single laboratory, a contribution of preanalytical heterogeneity cannot be fully excluded—reassuringly, block age showed no significant association with marker positivity (see Results). Whether pAMPK/pACC-negative samples harbor distinct genetic or signaling features remains a subject for future investigation. Notably, in our cohort, neither pAMPK nor pACC showed a statistically significant univariable association with OS in the overall cohort, regardless of whether a pre-set 5% or median expression value as a cut-off was employed.

We also have to note that the pAMPK-negative cohort was exceedingly small (n = 8), and structurally concentrated entirely within the regorafenib treatment arm. Because the comparator arm contained zero pAMPK-negative cases, biomarker status and treatment assignment are perfect confounders in this subset, preventing a true, treatment-independent prognostic comparison for this cut-point.

We hypothesize that GBM samples with pACC/pAMPK-positive expression may reflect elevated levels of upstream oncogenic signaling. Given that a substantial proportion of this signaling is mediated by receptor tyrosine kinases (RTKs)—indirectly supported by the estimated ~90% prevalence of RTK-linked alterations in GBM [16]—we hypothesized that pACC-positive tumors might be more sensitive to regorafenib, a multi-kinase inhibitor targeting RTK-driven signaling. This remains a molecular hypothesis that was not directly tested in the present study, which did not include RTK genomic or expression profiling.

In our previous study of 88 patients, we indeed observed that pACC expression correlated with improved response to regorafenib compared to lomustine [6]. In this real-world cohort, pACC expression > 5% was associated with longer OS in regorafenib-treated patients. However, the treatment-by-pACC interaction test for OS was non-significant. Statistical evaluation within a biomarker-high subgroup alone is insufficient to establish predictive capacity [17]. Consequently, pACC cannot be categorized as an actionable predictive biomarker based on these data. This study must be interpreted as a retrospective, hypothesis-generating analysis, and pACC should be viewed as an exploratory candidate requiring prospective interaction-powered testing [18]. This discrepancy may be attributed to the fact that, unlike the original REGOMA cohort, this real-world study observed a modest survival advantage for regorafenib even in pACC-negative tumors. Moreover, this statistical outcome may be partially due to the low number of pACC-negative (and even fewer pAMPK-negative) samples compared to our initial study [6]. Hypothetically, methodological refinements—including changes in the primary anti-pAMPK antibody and extended incubation times—likely increased staining sensitivity, thus resulting in a substantially lower percentage of pACC-negative samples despite using the same cut-off. At the same time, the observation that pACC-positive and pACC-negative tumors showed nearly identical OS in the fotemustine/lomustine-treated arm (6.4 vs 6.0 months) may be worth noting, although it should be interpreted with caution and does not, in itself, establish predictive biomarker status.

A further limitation of our study concerns the temporal relationship between biomarker assessment and clinical outcome. pACC expression was evaluated on FFPE tissue obtained at first surgery, whereas OS and PFS were calculated from the start of second-line treatment with regorafenib at recurrence, the time point at which the two treatment arms diverged following a uniform first-line regimen (surgery plus concomitant and adjuvant radio-chemotherapy according to the Stupp protocol [19]). This design reflects practical constraints, since tissue from first surgery was uniformly available for the whole cohort, whereas recurrent tissue could only be obtained in the subset of patients undergoing reoperation. However, it also means that pACC was measured as an archival, primary-tumor biomarker rather than as a marker of pathway activation immediately preceding second-line treatment.

This gap is biologically relevant. Longitudinal studies of IDH-wild-type glioblastoma have shown that primary and recurrent tumors frequently diverge at the genomic and epigenomic level, with several molecular alterations acquired or lost between diagnosis and recurrence under the selective pressure of radiotherapy and temozolomide [20]. Such molecular instability could plausibly extend to pathway activation states such as pACC expression, and may help explain the pattern observed in our cohort. Specifically, if the metabolic/kinase pathway status captured by pACC at first surgery does not fully reflect the biology of the recurrent, regorafenib-treated tumor, its correlation with PFS—a relatively proximal readout of the tumor’s response to treatment at recurrence—could be attenuated. Conversely, an association with OS may still emerge if baseline pACC also captures more stable, prognostically relevant features of the patient’s overall tumor biology and clinical trajectory (including response to subsequent lines of therapy), which are not fully mirrored by radiological progression alone. This could also contribute to the non-significant interaction test observed for OS, which may in part reflect limited statistical power in addition to biological heterogeneity. Studies incorporating paired primary–recurrent sampling will be needed to determine whether pACC expression remains stable across the disease course or evolves at recurrence, and to establish whether biomarker assessment at recurrence would improve its predictive value for treatment response.

Recently, the AGILE clinical trial failed to confirm a survival advantage for regorafenib over standard care (lomustine) in relapsed GBM [21]. While the AGILE authors suggested that the positive results of the REGOMA study might have been influenced by a lower-than-expected survival in the control arm, it is noteworthy that no biomarker assessment was conducted in the AGILE study.

In this regard, two observations are worth discussing. First, in our cohort, patients treated with regorafenib as second-line therapy achieved a significantly longer OS compared to those treated with fotemustine or lomustine (median OS: 10.4 months, 95% CI: 8.6–13.3 vs. 6.3 months, 95% CI: 5.4–7.7; log-rank *p* = 0.0021), as shown by univariate analysis. Importantly, while our study is retrospective and non-randomized—unlike the prospective randomized design of the REGOMA trial [5]—the OS observed in the control arm of our cohort (fotemustine/lomustine-treated patients, median OS: 6.3 months) is consistent with that of the lomustine arm in the REGOMA study (median OS: 5.6 months), arguing against an inflated control survival as a source of bias in our dataset. Second, given that our cohort was not prospectively selected, it is possible that it is enriched for patients with molecular features associated with regorafenib responsiveness, which could at least partly account for the favorable outcomes observed in the regorafenib-treated group.

In conclusion, this real-world cohort does not replicate the formal treatment-by-pACC statistical interaction signal for overall survival that was originally observed in the prospective REGOMA biomarker trial. Although regorafenib was associated with longer median OS in pACC-positive tumors, subgroup significance alone cannot establish predictive effect modification. This discrepancy—potentially related to differences in assay sensitivity or cohort composition—indicates that the predictive value of pACC cannot be considered formally established based on current evidence.

## 4. Material and Methods

### 4.1. Patient Samples

This retrospective translational study was approved by the institutional review board at the Ethics Committee for clinical experimentation (CESC) of Veneto Institute of Oncology (decision no. Em.2023-49 IOV-GB-1-2020-REGOMA-OSS issued on 04 April 2023) and at the Ethic Committee Area 2 Puglia (decision no. Em.2024-Prot.42548 issued on 15 May 2024) and conducted in accordance with the Declaration of Helsinki and Good Clinical Practice guidelines. Written informed consent, based on a form approved by the ethics committee of the enrolling institution in compliance with national regulations, was obtained from all patients. The present study included 174 archival tumor specimens, comprising 50 cases from the REGOMA-OSS cohort and 124 cases from a separate institutional series. No patient from the original randomized REGOMA biomarker cohort was included in this analysis. Patient characteristics are listed in Table 1.

### 4.2. In Vitro Studies with GBM Cell Lines

LN229 (CRL-2611, ATCC, Manassas, VA, USA) and U87-MG (HTB-14, ATCC, Manassas, VA, USA) cells were maintained in Dulbecco’s modified Eagle’s medium (DMEM) supplemented with 100 U/mL penicillin, 100 μg/mL streptomycin, 2 mM L-glutamine, and 10% heat-inactivated fetal calf serum (Gibco) under standard culture conditions. To assess the specificity of the monoclonal antibody (mAb) relative to the polyclonal antibody, LN229 cells were plated one day before transfection at a density of 2.5 × 10^5^ cells per well in six-well plates. On the following day, *ACACA* or *ACACB* silencing was performed using Lipofectamine RNAiMAX (Thermo Fisher Scientific) and *ACACA* siRNA (Ambion by Thermo Fisher Scientific, ID S883, cat. no. 4392420) or *ACACB* siRNA (Ambion by Thermo Fisher Scientific, ID S886, cat. no. 4392420), according to the manufacturer’s instructions.

After 48 h, cells were harvested, and phospho-ACC expression was analyzed by Western blot using both the monoclonal anti-phospho-ACC (Ser79, Cell Signaling Technology, cat. no. 11818) and the polyclonal anti-phospho-ACC antibody (Ser79, Cell Signaling Technology, cat. no. 3661). GAPDH was used as the loading control (GeneTex, cat. no. 100118). Detection was performed by ECL and visualized using a UVITEC imaging system.

To produce staining controls, U87-MG cells were transfected with either non-targeting control siRNA (Ambion by Thermo Fisher Scientific, cat. no. 4390843) or *ACACA* siRNA. After 48 h, the transfected cells were then processed to generate cell blocks [22], which were used as positive and negative controls for phospho-ACC staining, respectively.

### 4.3. Preanalytical Handling and Staining

All FFPE blocks were retrieved from the diagnostic archives of the participating centers and had been collected, fixed, and processed according to the standard diagnostic protocols in use at each institution at the time of surgery; centralized preanalytical records (cold ischemia time, time to fixation, fixation duration) were not available for this retrospective multicenter cohort, consistent with routine diagnostic archiving practice. All blocks were stored at room temperature. Block age spanned surgeries performed between 2007 and 2025 (n = 174; Supplementary Table S2), with the majority (83%, 144/174) collected between 2018 and 2023.

Immunohistochemistry (IHC) was performed on 4 μm thick FFPE tumor sections obtained at first surgery in all cases. Sections were cut 24 h prior to staining and dried overnight at room temperature to minimize epitope degradation, dewaxed using Bond Dewax Solution (Leica Microsystems, cat. no. AR9222), and rehydrated through a graded series of decreasing alcohol concentrations. To evaluate phosphorylated ACC (pACC) and phosphorylated AMPK (pAMPK) expression, separate sections were incubated for 1 h with one of the following primary antibodies: the monoclonal anti-phospho-ACC antibody (1:100, Ser79, Cell Signaling Technology, cat. no. 11818), the polyclonal anti-phospho-ACC antibody (1:50, Ser79, Cell Signaling Technology, cat. no. 3661), or the monoclonal anti-phospho-AMPK (1:100, Thr172, Cell Signaling Technology, cat. no. 2535). Antigen retrieval was carried out with citric acid buffer (pH 6.0, 10 mmol/L) in all samples. IHC was run on a Leica Autostainer Bond RX using the Bond Polymer Refine Detection Kit (Leica Microsystems, cat. no. DS9800). Immunostaining was revealed by incubation with 3,3-diaminobenzidine tetrahydrochloride hydrate for 10 min, followed by hematoxylin counterstaining for 5 min. As a positive control, a U87-MG cell block section was included on every slide. IHC staining was performed in a single laboratory; because of the size of the cohort, staining was carried out across multiple batches over time rather than in a single session.

### 4.4. Image Acquisition and Analysis

Slides were scanned at 200× magnification using the Aperio CS2 (Leica Biosystems), and IHC scoring was determined with Scanscope Image Analysis Software (ImageScope v12.4.2.5010) using Cytoplasmic algorithm v2 for pACC and pAMPK.

An Aperio Genie Classifier was trained to distinguish tumor tissue (foreground) from stroma, necrosis, vessels, non-neoplastic cells and glass (all grouped as background), and was then integrated with the Cytoplasmic v2 algorithm. Training was performed using 25 regions of interest (ROIs) per category, annotated on a single slide from the study cohort, selected as representative of the histological composition and staining variability of the series; this slide contributed a minor fraction of ROIs relative to the total tissue area subsequently evaluated across the cohort, limiting the risk of overfitting. Classifier performance was evaluated on an extended tissue area of the training slide, following standard digital pathology validation principles [23], as per the manufacturer’s protocol, yielding a sensitivity/specificity of 99.1%/87.3% for tumor tissue detection and 94.86%/99.66% for background detection. Tumor area recognition by the classifier was further checked against pathologist assessment on 20 hematoxylin–eosin (H&E) sections contiguous to those used for IHC, in which a pathologist independently outlined and estimated the percentage of tumor area; this qualitative comparison showed good agreement with classifier-based segmentation, supporting the reliability of tissue recognition, although a formal concordance statistic was not calculated. Threshold calibration and overall supervision of the digital analysis were performed by F.F., who has specific experience with this digital pathology software. Tumor regions were segmented automatically by the classifier; the analyzed area was manually restricted only to exclude tissue artifacts and sectioning imperfections not reliably recognized by the algorithm. The Cytoplasmic v2 algorithm classifies each segmented cell based on the optical density of the IHC signal in the relevant chromogenic channel, using an inverse 0–255 grayscale intensity scale, where 0 corresponds to maximal staining intensity (black) and 255 to absence of staining (white); lower values therefore indicate more intense staining. Cytoplasmic v2 intensity thresholds were set at 195 (1+), 180 (2+), and 140 (3+) intensity units, with an upper cell-detection threshold of 210. These values differed from those used in our previous REGOMA cohort [6] (200/210/165, upper threshold 221), reflecting the overall higher staining intensity observed in the current series; no other analytical parameters differed between the two workflows. The output provided the percentage of cells with protein expression categorized as 3+ (highly positive), 2+ (intermediate positive), 1+ (low positive), and 0 (negative), with the sum of these four categories equaling 100% for each sample. For statistical analysis, the percentages of cells with 0/1+ and 2+/3+ expression were pooled within each sample. Samples were classified as pACC-negative (≤5% of tumor cells with 2+/3+ staining) or pACC-positive (>5%), and similarly for pAMPK, as in our previous study [6]. All digital scoring was performed blinded to treatment arm, clinical outcome, and biomarker group. Although this is a multicenter study, all IHC staining was performed at a single center across multiple staining runs due to cohort size; staining reproducibility was monitored using an internal control (a U87-MG cell block section) included on every slide processed.

Spatial expression patterns of pACC- and pAMPK-positive samples were classified as peri-necrotic (positive cells concentrated within a rim adjacent to necrotic areas), peri-vascular (positive cells clustered around vascular structures), homogeneous (diffuse, uniform distribution of positive cells throughout the tumor section), or mixed (coexistence of two or more of the above patterns within the same section). Pattern assignment was performed by a single observer (F.F.), blinded to clinical outcome and treatment arm, based on visual inspection of whole-slide images. As spatial pattern classification was performed by a single blinded observer, interobserver reproducibility could not be assessed; this represents a limitation of the study, and future work should include independent scoring by a second observer to formally evaluate concordance.

### 4.5. Statistical Analysis

The primary aim was to externally validate the role of pACC as a predictive marker of overall survival in a prospective cohort of recurrent glioblastoma patients treated with regorafenib in the REGOMA-OSS cohort [7] trial and in retrospective/prospective cohorts of recurrent glioblastoma patients treated with chemotherapy. According to previous results [6], the proportion of patients who died of disease was 83% and in around 64% of patients, the biomarker levels were high. With the above assumptions, a sample of 144 patients (1:1 design; 72 patients treated with regorafenib, 72 patients treated with chemotherapy) allows to estimate a hazard ratio for the interaction between treatment and biomarker levels equal to 0.34, with 80% power and a 5% significance level (two-sided) [24]. Secondarily, we aimed to evaluate the predictive role of pAMPK and to estimate the association of treatment and markers with overall survival (OS) and progression-free survival (PFS). OS was defined as the time from the start of second-line treatment to death from any cause; PFS was defined as the time from the start of second-line treatment to documented disease progression according to Response Assessment in Neuro-Oncology (RANO) criteria or death from any cause, whichever occurred first. Follow-up brain MRI scans were routinely performed every 8 to 12 weeks according to standard clinical practice. Patients remaining alive and without documented progression were censored at the date of last objective neuro-imaging evaluation. Due to high event maturity (94.3% mortality), reverse Kaplan–Meier estimation was heavily right-truncated; follow-up duration was therefore summarized as median and interquartile range among surviving (censored) patients.

The predictive role of baseline pACC or pAMPK expression was estimated including a multiplicative interaction term with treatment in the Cox proportional hazards model to formally test if relative treatment effects varied significantly by biomarker status. Results were reported as hazard ratios with the 95% confidence interval (CI).

To evaluate OS while accounting for non-randomized treatment assignment and baseline covariate imbalances, both multivariable Cox proportional hazards regression and Inverse Probability of Treatment Weighting (IPTW) were implemented [25,26]. Covariates included in adjusted models and propensity score estimation (age, sex, ECOG performance status, and MGMT promoter methylation status) were selected a priori as they represent universally recognized, standard clinical and molecular prognostic indicators in recurrent IDH-wild-type glioblastoma.

Recognizing a structural violation of the positivity assumption in the full cohort—specifically, the exclusive assignment of patients with poorer performance status (ECOG PS 2–3) to the control arm—we restricted the IPTW-adjusted framework to the subset of patients with ECOG PS 0–1 (N = 136). This restriction guaranteed strict common support and prevented reliance on model-based extrapolation. Comprehensive methodological details, covariate balance diagnostics (Love plots and density overlap plots, Figure S7), and the pooled survival models for the IPTW approach are provided in the Supplementary Materials.

Missing covariate data were addressed using Multiple Imputation by Chained Equations (MICE) with M = 20 imputed datasets [27].

A further sensitivity analysis was also conducted to evaluate marker cut-offs other than those selected a priori, using the median expression value as an alternative dichotomization threshold.

Baseline demographic and clinical characteristics were summarized by treatment arm. Continuous variables were reported as medians with interquartile ranges (Q1, Q3), and categorical variables were presented as frequencies and percentages. Comparisons between groups were performed using the Kruskal–Wallis, chi-square or Fisher’s exact test, as appropriate.

Relationships between the expression levels of pAMPK, pACC, and ACC were assessed using Spearman’s rank correlation coefficient, alongside 95% confidence intervals (CIs) and *p*-values.

Survival probabilities were estimated using the Kaplan–Meier method, and summarized as median times with 95% CIs, calculated using the Brookmeyer–Crowley method, as well as survival probabilities at specific time points with corresponding 95% CIs. Survival distributions between treatment arms and biomarker groups were compared using the log-rank test. Survival probabilities were estimated using the Kaplan–Meier method, compared using the log-rank test, and summarized as median times with 95% CIs, calculated using the Brookmeyer–Crowley method. Formal adjustments for multiplicity were not performed across secondary and exploratory endpoints.

All statistical analyses were conducted using R version 4.4.2.

## 5. Conclusions

Overall, this study expands current knowledge on AMPK signaling in GBM by demonstrating widespread but heterogeneous pACC and pAMPK expression in a large real-world cohort. While neither marker showed a statistically significant univariable association with OS in the overall cohort, pACC-positive status was associated with better outcomes among regorafenib-treated patients, consistent with the association observed in the original REGOMA trial [6]. However, unlike in that trial, the formal treatment-by-pACC interaction test did not reach statistical significance in the present cohort, and pACC therefore cannot currently be considered a validated predictive biomarker. Taken together, these findings support further investigation of pACC and pAMPK in prospective studies with adequate statistical power to formally test for treatment effect modification, before any biomarker-based patient selection strategy can be considered.

## Figures and Tables

**Table 1:** Baseline characteristics of patients analyzed for pACC and pAMPK expression.

Glioblastoma n = 174		Fotemustine/ Lomustine (N = 90)	Regorafenib (N = 84)	*p*-Value
**Age**	Median (Q1, Q3)	63.5 (55.4, 70.4)	60.6 (53.2, 65.9)	0.112
**Gender**	Male	63 (70.0%)	47 (56.0%)	0.055
	Female	27 (30.0%)	37 (44.0%)	
**ECOG PS**	N-Miss	19	9	<0.001 *
	0–1	61 (85.9%)	75 (100.0%)	
	2–3	10 (14.1%)	0 (0.0%)	
**Target Hemisphere**	N-Miss	10	7	0.308
	Right	33 (41.2%)	38 (49.4%)	
	Left	47 (58.8%)	39 (50.6%)	
**Target Lobes**	N-Miss	21	10	0.936 *
	Temporal	29 (42.0%)	30 (40.5%)	
	Occipital	3 (4.3%)	4 (5.4%)	
	Parietal	14 (20.3%)	14 (18.9%)	
	Frontal	22 (31.9%)	23 (31.1%)	
	Other	1 (1.4%)	3 (4.1%)	
***MGMT***	N-Miss	8	10	0.446
	Non-methylated	36 (43.9%)	37 (50.0%)	
	Methylated	46 (56.1%)	37 (50.0%)	
**pACC**	Median (Q1, Q3)	46.5 (20.3, 68.7)	26.2 (9.0, 56.4)	***0.009***
**pAMPK**	Median (Q1, Q3)	71.2 (50.5, 86.8)	53.0 (27.5, 80.1)	***0.004***

* Fisher test.

**Table 2:** Univariable and multivariable Cox regression analysis for overall survival by treatment arm and metabolic biomarker expression (pACC and pAMPK).

		n	Events	Median OS (95%CI)	HR (95%CI)	*p-Value*	Adjusted ‡ HR (95%CI)	*p-Value*
**Treatment**	Fotemustine/ Lomustine	90	88	6.3 (5.4, 7.7)	Ref		Ref	
	Regorafenib	84	76	10.4 (8.6, 13.3)	0.62 (0.45, 0.84)	***0.0023***	0.69 (0.49, 0.97)	***0.0344***
**pACC**	Negative (≤5.0%)	25	24	7.5 (5.0, 12.2)	Ref		Ref	
	Positive (>5.0%)	149	140	8.3 (7.3, 9.4)	0.86 (0.56, 1.33)	*0.4970*	1.02 (0.6, 1.72)	*0.9516*
**pAMPK**	Negative (≤5.0%)	8	7	7.0 (2.6, 14.5)	Ref		Ref	
	Positive (>5.0%)	166	157	8.1 (7.0, 9.1)	0.79 (0.37, 1.69)	*0.5390*	0.73 (0.30, 1.76)	*0.4776*

Note: Hazard ratios (HR) and *p*-values were derived from univariable and multivariable Cox proportional hazards regression models; statistically significant *p*-values (*p* < 0.05) are shown in red bold italic. ‡ Multivariable Cox model including treatment, age, gender, MGMT methylation, and biomarker expression levels (pACC and pAMPK).

## Data Availability

The raw immunohistochemical quantification data for pAMPK and pACC expression presented in the study are openly available in Zenodo repository at https://doi.org/10.5281/zenodo.20282645, released under a Creative Commons Attribution 4.0 International (CC BY 4.0) license. Other data generated or analyzed during this study are included in this article or in the Supplementary Materials accompanying this paper.

## Supplementary Materials

The following supporting information can be downloaded at: https://www.mdpi.com/article/10.3390/ijms27177789/s1.

## References

[1] Sahai V., Rothe M., Mangat P.K., Garrett-Mayer E., Suhag V., Dib E.G., Mehmi I., Kadakia K.C., Pisick E., Duvivier H.L. (2024). Regorafenib in Patients With Solid Tumors With *BRAF* Alterations: Results From the Targeted Agent and Profiling Utilization Registry (TAPUR) Study. JCO Precis. Oncol..

[2] Wilhelm S.M., Dumas J., Adnane L., Lynch M., Carter C.A., Schütz G., Thierauch K., Zopf D. (2011). Regorafenib (BAY 73-4506): A New Oral Multikinase Inhibitor of Angiogenic, Stromal and Oncogenic Receptor Tyrosine Kinases with Potent Preclinical Antitumor Activity. Int. J. Cancer.

[3] Daudigeos-Dubus E., Le Dret L., Lanvers-Kaminsky C., Bawa O., Opolon P., Vievard A., Villa I., Pagès M., Bosq J., Vassal G. (2015). Regorafenib: Antitumor Activity upon Mono and Combination Therapy in Preclinical Pediatric Malignancy Models. PLoS ONE.

[4] Mongiardi M.P., Pallini R., D’Alessandris Q.G., Levi A., Falchetti M.L. (2024). Regorafenib and Glioblastoma: A Literature Review of Preclinical Studies, Molecular Mechanisms and Clinical Effectiveness. Expert Rev. Mol. Med..

[5] Lombardi G., De Salvo G.L., Brandes A.A., Eoli M., Rudà R., Faedi M., Lolli I., Pace A., Daniele B., Pasqualetti F. (2019). Regorafenib Compared with Lomustine in Patients with Relapsed Glioblastoma (REGOMA): A Multicentre, Open-Label, Randomised, Controlled, Phase 2 Trial. Lancet Oncol..

[6] Indraccolo S., De Salvo G.L., Verza M., Caccese M., Esposito G., Piga I., Del Bianco P., Pizzi M., Gardiman M.P., Eoli M. (2020). Phosphorylated Acetyl-CoA Carboxylase Is Associated with Clinical Benefit with Regorafenib in Relapsed Glioblastoma: REGOMA Trial Biomarker Analysis. Clin. Cancer Res..

[7] Caccese M., Desideri I., Villani V., Simonelli M., Buglione M., Chiesa S., Franceschi E., Gaviani P., Stasi I., Caserta C. (2024). REGOMA-OSS: A Large, Italian, Multicenter, Prospective, Observational Study Evaluating the Efficacy and Safety of Regorafenib in Patients with Recurrent Glioblastoma. ESMO Open.

[8] Lorenz N.I., Sauer B., Urban H., Weinem J.-B., Parmar B.S., Zeiner P.S., Strecker M.I., Schulte D., Mittelbronn M., Alekseeva T. (2025). AMP-Activated Protein Kinase Mediates Adaptation of Glioblastoma Cells to Conditions of the Tumor Microenvironment. J. Exp. Clin. Cancer Res..

[9] Jones J.E.C., Esler W.P., Patel R., Lanba A., Vera N.B., Pfefferkorn J.A., Vernochet C. (2017). Inhibition of Acetyl-CoA Carboxylase 1 (ACC1) and 2 (ACC2) Reduces Proliferation and De Novo Lipogenesis of EGFRvIII Human Glioblastoma Cells. PLoS ONE.

[10] Wang Y., Yu W., Li S., Guo D., He J., Wang Y. (2022). Acetyl-CoA Carboxylases and Diseases. Front. Oncol..

[11] Laderoute K.R., Amin K., Calaoagan J.M., Knapp M., Le T., Orduna J., Foretz M., Viollet B. (2006). 5′-AMP-Activated Protein Kinase (AMPK) Is Induced by Low-Oxygen and Glucose Deprivation Conditions Found in Solid-Tumor Microenvironments. Mol. Cell. Biol..

[12] Liang J., Mills G.B. (2013). AMPK: A Contextual Oncogene or Tumor Suppressor?. Cancer Res..

[13] Chhipa R.R., Fan Q., Anderson J., Muraleedharan R., Huang Y., Ciraolo G., Chen X., Waclaw R., Chow L.M., Khuchua Z. (2018). AMP Kinase Promotes Glioblastoma Bioenergetics and Tumour Growth. Nat. Cell Biol..

[14] Rong Y., Durden D.L., Van Meir E.G., Brat D.J. (2006). ‘Pseudopalisading’ Necrosis in Glioblastoma: A Familiar Morphologic Feature That Links Vascular Pathology, Hypoxia, and Angiogenesis. J. Neuropathol. Exp. Neurol..

[15] Godlewski J., Nowicki M.O., Bronisz A., Nuovo G., Palatini J., De Lay M., Van Brocklyn J., Ostrowski M.C., Chiocca E.A., Lawler S.E. (2010). MicroRNA-451 Regulates LKB1/AMPK Signaling and Allows Adaptation to Metabolic Stress in Glioma Cells. Mol. Cell.

[16] Qin A., Musket A., Musich P.R., Schweitzer J.B., Xie Q. (2021). Receptor Tyrosine Kinases as Druggable Targets in Glioblastoma: Do Signaling Pathways Matter?. Neurooncol. Adv..

[17] Polley M.-Y.C., Freidlin B., Korn E.L., Conley B.A., Abrams J.S., McShane L.M. (2013). Statistical and Practical Considerations for Clinical Evaluation of Predictive Biomarkers. JNCI J. Natl. Cancer Inst..

[18] Ballman K.V. (2015). Biomarker: Predictive or Prognostic?. J. Clin. Oncol..

[19] Stupp R., Mason W.P., Van Den Bent M.J., Weller M., Fisher B., Taphoorn M.J.B., Belanger K., Brandes A.A., Marosi C., Bogdahn U. (2005). Radiotherapy plus Concomitant and Adjuvant Temozolomide for Glioblastoma. N. Engl. J. Med..

[20] Draaisma K., Chatzipli A., Taphoorn M., Kerkhof M., Weyerbrock A., Sanson M., Hoeben A., Lukacova S., Lombardi G., Leenstra S. (2020). Molecular Evolution of *IDH* Wild-Type Glioblastomas Treated with Standard of Care Affects Survival and Design of Precision Medicine Trials: A Report From the EORTC 1542 Study. J. Clin. Oncol..

[21] Wen P.Y., Berry D.A., Buxton M.B., Colman H., de Groot J., Lim M., Mellinghoff I., Perry J.R., Weller M., Blondin N.A. (2026). Evaluation of Regorafenib in Newly Diagnosed and Recurrent Glioblastoma: GBM AGILE Phase II/III Bayesian Randomized Platform Trial. J. Clin. Oncol..

[22] Saharti S. (2024). Contemporary Art of Cell-Block Preparation: Overview. Cytojournal.

[23] Aeffner F., Zarella M.D., Buchbinder N., Bui M.M., Goodman M.R., Hartman D.J., Lujan G.M., Molani M.A., Parwani A.V., Lillard K. (2019). Introduction to Digital Image Analysis in Whole-Slide Imaging: A White Paper from the Digital Pathology Association. J. Pathol. Inform..

[24] Schmoor C., Sauerbrei W., Schumacher M. (2000). Sample Size Considerations for the Evaluation of Prognostic Factors in Survival Analysis. Stat. Med..

[25] Cole S.R., Hernán M.A. (2004). Adjusted Survival Curves with Inverse Probability Weights. Comput. Methods Programs Biomed..

[26] Austin P.C., Stuart E.A. (2015). Moving towards Best Practice When Using Inverse Probability of Treatment Weighting (IPTW) Using the Propensity Score to Estimate Causal Treatment Effects in Observational Studies. Stat. Med..

[27] Buuren S.V., Groothuis-Oudshoorn K. (2011). Mice: Multivariate Imputation by Chained Equations in R. J. Stat. Softw..

