# Supplementary material for: AMPK Pathway Activation Markers in GBM: Expression Patterns and Clinical Associations in a Real-World Study
Source: Int J Mol Sci. 2026 Aug 31;27(17):7789. doi: 10.3390/ijms27177789 (PMC13566083; doi:10.3390/ijms27177789)
Supplement: Supplementary file 1 [file ijms-27-07789-s001.zip › SUPPLEMENTARY MATERIAL Ferrarini Del Bianco_REVISED FF PDB (rev4) CLEAR.pdf]

AMPK pathway activation markers in GBM: expression patterns and clinical associations in a real-world study

SUPPLEMENTARY MATERIAL

Federica Ferrarini [1,#], Paola Del Bianco [2,#], Martina Corrà [3], Tiziana Talienti [4], Matteo Mauceri [1], Daniele Boso [1], Giusi Romanazzi [4], Martina Bedeschi [5], Mario Caccese [3], Marta Padovan [3], Angela Guerriero [6], Giovanni Esposito [7], Isacco Desideri [8,9], Enrico Franceschi [10], Paola Gaviani [11], Michela Buglione di Monale e Bastia [12], Gian Luca De Salvo [2], Alba Fiorentino [4,13], Anna Tesei [5], Tommaso Mazza [14,$], Giuseppe Lombardi [3] and Stefano Indraccolo [1,15,*]

Supplementary results
To ensure the validity and stability of results against potential confounding by indication and selection bias due to missing data, we evaluated the treatment effect across the statistical configurations detailed above.

In the univariable analysis utilizing all available information for each variable, second-line treatment with regorafenib was associated with a significant reduction in the risk of death compared to fotemustine/lomustine (HR = 0.62; 95% CI: 0.45–0.84; p = 0.002).

To reduce the risk of model-based extrapolation caused by the baseline ECOG imbalance, adjusted survival models were restricted to the common-support cohort (ECOG 0–1; N = 136). To limit the loss of statistical power due to missing data within this subset, models were pooled across multiply-imputed datasets. Accounting for baseline selection bias using the pooled IPTW-adjusted model confirmed that the survival benefit remained robustly significant. The pooled IPTW-adjusted HR for regorafenib was 0.65 (95% CI: 0.44–0.95; p = 0.025) (Table S3).

This finding was fully corroborated by the final pooled multivariable Cox model, where regorafenib was confirmed as an independent predictor of superior overall survival after adjusting for covariates (adjusted HR = 0.57; 95% CI: 0.45–0.82; p = 0.003) (Table S3).

Taken together, these rigorous sensitivity analyses indicate that, after enforcing treatment overlap, adjusting for key prognostic confounders and addressing missing data, second-line regorafenib was associated with a statistically significant independent reduction in the risk of death compared with fotemustine/lomustine.

Supplementary materials and methods
This supplementary section outlines the statistical frameworks utilized to validate the primary overall survival (OS) findings. Because retrospective real-world studies are susceptible to missing data and confounding by indication (e.g., the selective assignment of healthier patients to a specific treatment arm), we implemented parallel causal-inference configurations to confirm the stability of the treatment effect.

Statistical Analysis

Handling of Missing Data

Missing covariate data were addressed using Multiple Imputation by Chained Equations (MICE) under the missing-at-random (MAR) assumption. Imputation models incorporated all baseline clinical covariates, biomarker expression (pACC and pAMPK), event indicators, and the cumulative hazard function. A total of M = 20 imputed datasets were generated.

Propensity Score Estimation and Adjustments

To address non-randomized treatment assignment and baseline performance status imbalances (specifically, the exclusive presence of patients with ECOG PS 2–3 in the alkylating control arm), causal inference models were implemented on a restricted common-support cohort. Restricting propensity score estimation and weighting strictly to patients with ECOG PS 0–1 (N = 136) eliminated structural positivity violations and prevented model-based extrapolation. Propensity scores estimating the probability of receiving regorafenib were calculated using logistic regression based on age, gender, and MGMT promoter methylation status. Stabilized IPTW weights were applied, and balance was evaluated using standardized mean differences (SMD), with SMD < 0.10 indicating optimal balance across all covariates. Both pooled IPTW Cox proportional hazards models and multivariable Cox regression models were fitted across this restricted cohort (Figures S6 and S7).

Survival Analysis Frameworks

Overall survival (OS) was evaluated from the initiation of second-line therapy using Cox proportional hazards regression models. To demonstrate the stability of the treatment effect, survival models were structured across two distinct frameworks:

Unadjusted Available Data Framework (Pairwise Available-Case Analysis):

1. *Univariable Cox Models:* Crude models assessing the unadjusted effect of the second-line drug and baseline covariates, utilizing all available non-missing data for each respective variable (ranging up to N = 174).

Restricted Common-Support Framework (ECOG 0-1, Imputed, Data Framework (Full Cohort, N = 136): To strictly satisfy the positivity assumption while limiting the loss of statistical power via MICE imputation, adjusted models were executed on the restricted cohort.

1. *Pooled IPTW-Adjusted Model:* Survival models executed across the weighted, fully imputed datasets.
2. *Pooled Multivariable Cox Model:* A fully adjusted multivariable regression model executed and pooled across the imputed datasets.

Statistical estimates, including Hazard Ratios (HR) and 95% Confidence Intervals (CI) from the imputed datasets, were combined using Rubin's rules to produce the final pooled results. All analyses were performed using R software (version 4.4.2).

Supplementary Tables

Supplementary Table S1. Baseline characteristics of patient analyzed for pACC and pAMPK expression in the fotemustine and lomustine control groups.

		Fotemustine (N=66)	Lomustine (N=24)	*p-value*
**Age**	Median (Q1, Q3)	63.5 (53.0, 70.6)	63.5 (59.3, 67.6)	0.695
**Gender**	Male	47 (71.2%)	16 (66.7%)	0.796
	Female	19 (28.8%)	8 (33.3%)	
**ECOG PS**	N-Miss	17	2	
	0 - 1	46 (93.9%)	15 (68.2%)	***0.008‡***
	2 - 3	3 (6.1%)	7 (31.8%)	
**Target Hemisphere**	NMiss	7	3	
	Right	27 (45.8%)	6 (28.6%)	0.204
	Left	32 (54.2%)	15 (71.4%)	
**Target Lobes**	NMiss	17	4	
	Temporal	22 (44.9%)	7 (35.0%)	
	Occipital	2 (4.1%)	1 (5.0%)	
	Parietal	8 (16.3%)	6 (30.0%)	0.752‡
	Frontal	16 (32.7%)	6 (30.0%)	
	Other	1 (2.0%)	0 (0.0%)	
**MGMT**	NMiss	8	0	
	Non-methylated	27 (46.6%)	9 (37.5%)	0.476
	Methylated	31 (53.4%)	15 (62.5%)	
**pACC (cont.)**	Median (Q1, Q3)	121.6 (66.0, 165.9)	138.6 (84.8, 173.5)	0.584
**pAMPK (cont.)**	Median (Q1, Q3)	188.5 (128.9, 227.0)	173.8 (146.6, 206.4)	0.541
**OS**	Median (Q1, Q3)	6.8 (3.9, 11.1)	6.2 (2.3, 8.7)	0.209*

*‡ Fisher test; *Log-rank test*

Supplementary Table S2. pACC and pAMPK positivity by block age

Block age (year)	n	pACC > 5%	pACC ≤ 5%	% pACC > 5%	pAMPK > 5%	pAMPK ≤ 5%	% pAMPK > 5%
2007	1	1	0	100.0%	0	1	0.0%
2011	1	1	0	100.0%	1	0	100.0%
2013	3	2	1	66.7%	3	0	100.0%
2014	5	5	0	100.0%	5	0	100.0%
2015	3	3	0	100.0%	3	0	100.0%
2016	9	9	0	100.0%	9	0	100.0%
2017	8	8	0	100.0%	8	0	100.0%
2018	11	11	0	100.0%	11	0	100.0%
2019	19	18	1	94.7%	18	1	94.7%
2020	33	28	5	84.8%	28	5	84.8%
2021	26	25	1	96.2%	25	1	96.2%
2022	30	30	0	100.0%	30	0	100.0%
2023	21	21	0	100.0%	21	0	100.0%
2024	3	3	0	100.0%	3	0	100.0%
2025	1	1	0	100.0%	1	0	100.0%
Total	174	166	8	95.4%	166	8	95.4%

Note: Cochran–Armitage trend test (positivity vs. block age):
pACC: z = 0.78, p = 0.438
pAMPK: z = 1.56, p = 0.119
No significant trend in marker positivity was observed as a function of block age for either pACC or pAMPK.

Supplementary Table S3. Comparison of survival analyses (Unadjusted Pairwise Available-Case vs. Complete-Case and Imputed/Weighted Models) for overall survival.

	Available data configurations		Restricted cohort (ECOG 0-1, N=136)			
	Univariable		Pooled IPTW-Adjusted*		Pooled Multivariable Cox**	
Variable	HR (95% CI)	*p-value*	HR (95% CI)	p-value	HR (95% CI)	p-value
**Treatment**: Regorafenib *(Ref: Fotemustine/Lomustine)*	0.62 (0.45 – 0.84)	***0.002***	0.64 (0.44 – 0.93)	***0.021***	0.54 (0.37, 0.79)	***0.002***
**Age** *(per additional year)*	1.03 (1.01 – 1.04)	***< 0.001***			1.04 (1.02, 1.06)	***< 0.001***
**Gender**: Female *(Ref: Male)*	0.80 (0.58 – 1.11)	*0.180*			0.77 (0.52, 1.12)	*0.170*
**MGMT Status**: Methylated *(Ref: Non-methylated)*	0.74 (0.53 – 1.04)	*0.079*			0.78 (0.53, 1.14)	*0.198*
**pACC**: Positive *(Ref: Negative)*	0.86 (0.56, 1.33)	*0.497*			0.91 (0.50, 1.67)	*0.762*
**pAMPK**: Positive *(Ref: Negative)*	0.79 (0.37, 1.69)	*0.539*			0.85 (0.33, 2.20)	*0.728*

Note: HR = Hazard Ratio; CI = Confidence Interval; IPTW = Inverse Probability of Treatment Weighting.
Univariable Analysis was conducted via a pairwise available-case approach maximizing the data present for each specific variable (Treatment: N=174; Age: N=174; Gender: N=174; MGMT status: N=156).

**Pooled Adjusted Models utilize the restricted ECOG 0–1 sample population (N = 136), imputed and pooled across m = 20 datasets. Propensity score models were conditioned on age, gender, and MGMT status. Covariates were selected a priori based on established neuro-oncological evidence.*

Supplementary Table S4. Univariable and multivariable Cox regression analysis for progression free survival by treatment arm and metabolic biomarker expression (pACC and pAMPK).

		n	events	median PFS (95%CI)	HR (95% CI)	*p-value*	Adjusted‡ HR (95% CI)	*p-value*
Treatment	Fotemustine/Lomustine	90	90	2.4 (2.3, 2.7)	Ref		Ref	
	Regorafenib	84	81	2.6 (2.2, 3.7)	0.85 (0.63;1.15)	0.3010	0.91 (0.66, 1.27)	0.5880
pACC	Negative	25	24	2.6 (2.3, 3.2)	Ref		Ref	
	Positive	149	147	2.4 (2.3, 3.0)	1.22 (0.79;1.88)	0.3770	1.17 (0.68, 2.00)	0.5633
pAMPK	Negative	8	7	5.0 (1.2, 12.2)	Ref		Ref	
	Positive	166	164	2.4 (2.3, 2.8)	1.51 (0.71;3.22)	0.2890	1.38 (0.54, 3.49)	0.4957

Note: Patients were stratified by treatment arm (Fotemustine/Lomustine vs. Regorafenib) and by immunohistochemical expression of pACC and pAMPK. For each subgroup, the number of patients (n), number of events, and median PFS with 95% confidence intervals (95%CI) are reported. Negative and Positive expression categories were defined using an immunohistochemical score cutoff of 5%. Hazard ratios (HR) and p-values were derived from univariable and multivariable Cox proportional hazards regression models. ‡ Multivariable Cox model including treatment, age, gender, MGMT methylation, and biomarker expression levels (pACC and pAMPK).

Supplementary figures

A

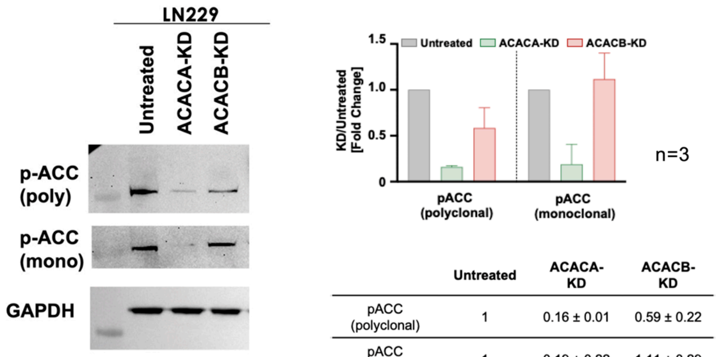

	Untreated	ACACA-KD	ACACB-KD
pACC (polyclonal)	1	0.16 ± 0.01	0.59 ± 0.22
pACC (monoclonal)	1	0.19 ± 0.22	1.11 ± 0.29

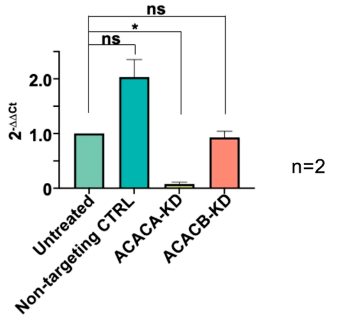

WB **RT-qPCR**

B

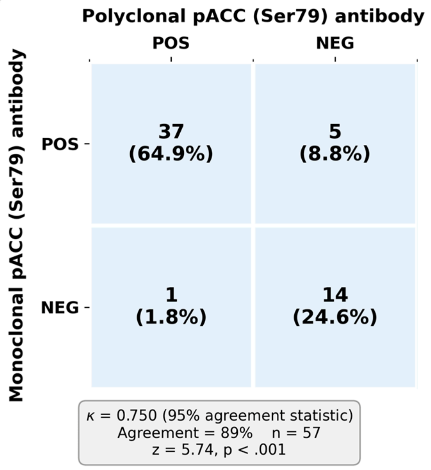

C

Days after slides preparation	Sample	Antibody	AVG	SD	LCI	UCI	CV
1	142-80	Monoclonal	7.31	3.69	-1.86	16.50	50.50
1	142-80	Polyclonal	4.16	3.67	-4.95	13.30	88.20
1	1763-6	Monoclonal	17.20	9.74	-6.96	41.40	56.50
1	1763-6	Polyclonal	13.00	3.26	4.92	21.10	25.10
1	1763-7	Monoclonal	72.10	5.49	58.40	85.70	7.62
1	1763-7	Polyclonal	72.10	2.27	51.70	92.60	3.15
5	142-80	Monoclonal	5.46	0.51	4.19	6.73	9.34
5	142-80	Polyclonal	2.13	0.85	0.01	4.25	40.10
5	1763-6	Monoclonal	10.50	1.94	5.66	15.30	18.50
5	1763-6	Polyclonal	13.40	5.05	0.81	25.90	37.80
5	1763-7	Monoclonal	57.70	15.10	20.20	95.20	26.20
5	1763-7	Polyclonal	69.80	3.91	60.10	79.60	5.60
7	142-80	Monoclonal	2.56	0.80	0.57	4.56	31.30
7	142-80	Polyclonal	2.66	1.47	-0.99	6.31	55.20
7	1763-6	Monoclonal	7.76	7.94	-12.00	27.50	102.00
7	1763-6	Polyclonal	12.10	1.00	9.57	14.60	8.33
7	1763-7	Monoclonal	74.30	21.50	20.90	128.00	28.90
7	1763-7	Polyclonal	54.70	34.30	-30.50	140.00	62.70

D

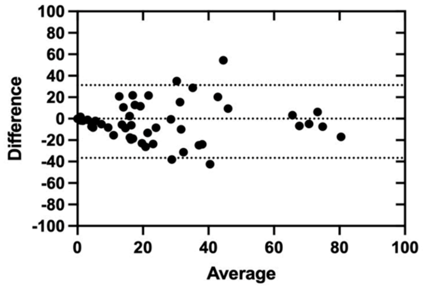

Supplementary Figure S1. Comparison of monoclonal and polyclonal anti-phospho-ACC antibodies.
(A) In the left panel, Western blot analysis of pACC expression in lysates of the LN229 cell line following lipofection of *ACACA*- or *ACACB*-specific siRNA (KD labels). The panel shows that the mAb (mono) is specific for the *ACACA*-encoded isoform, whereas the polyclonal Ab (poly) binds to both pACC isoforms. Bar graphs on the right show densitometric quantification of pACC monoclonal and polyclonal protein levels normalized to GAPDH, from three independent experiments. Data are expressed as fold change relative to the untreated condition (mean ± SD). Upon *ACACA* knockdown, pACC levels were markedly reduced (0.19 ± 0.22), while *ACACB* knockdown had no appreciable effect on pACC signal

(1.11 ± 0.29), confirming isoform specificity of the monoclonal antibody. In the right panel, relative mRNA expression levels of *ACACA* and *ACACB* in LN229 cells transfected with siRNA targeting *ACACA* or *ACACB*, compared with untreated and non-targeting control, as determined by real-time PCR and analyzed using the $2^{-\Delta\Delta Ct}$ method. *ACACA* siRNA markedly reduced *ACACA* expression, whereas *ACACB* siRNA did not alter *ACACA* levels. Importantly, the non-targeting control did not reduce *ACACA* expression, confirming that the decrease observed with *ACACA* siRNA is specific and not due to the transfection procedure. (B) Categorical agreement between monoclonal and polyclonal anti-pACC (Ser79) antibodies. IHC staining was performed on 57 GBM sections using both antibodies, and cytoplasmic positivity was quantified using a dedicated cytoplasmic algorithm in ImageScope software (Leica Biosystems). A cut-off of 5% positive cells was applied to define pACC positivity, in accordance with previously published criteria, as summarized in the 2×2 contingency table. Overall categorical agreement was 89.5% (51/57 concordant classifications), with a Cohen's κ of 0.75 (95% CI 0.56-0.94), indicating substantial agreement. Six samples (10.5%) were discordant across the threshold: five were positive with the monoclonal antibody only, and one was positive with the polyclonal antibody only. (C) Inter-run reproducibility of pACC (Ser79) immunostaining. Three representative GBM samples spanning the range of pACC positivity (142-80, 1763-6, 1763-7) were re-stained with the monoclonal and polyclonal anti-pACC antibodies on three separate staining runs, performed 1, 5 and 7 days after slide sectioning, and scored by a single observer using the same cytoplasmic algorithm as in panel B. Samples 1763-6 and 1763-7 were part of the present validation series, while sample 142-80 originated from the earlier REGOMA cohort [6]. For each sample/antibody/time-point combination, data are expressed as mean percentage positivity (AVG) ± standard deviation (SD), with the corresponding 95% confidence interval (LCI–UCI) and coefficient of variation (CV, %). Inter-run reproducibility was good (CV generally <30%) for samples with clearly high or unequivocally low positivity, whereas greater variability (CV up to ~100%) was observed for samples/time-points falling in the low-to-intermediate positivity range, consistent with the higher inter-antibody discordance observed near the 5% positivity threshold (panel B). (D) Concordance between the pACC expression values obtained with the two primary anti-pACC antibodies was also assessed by Bland–Altman analysis.

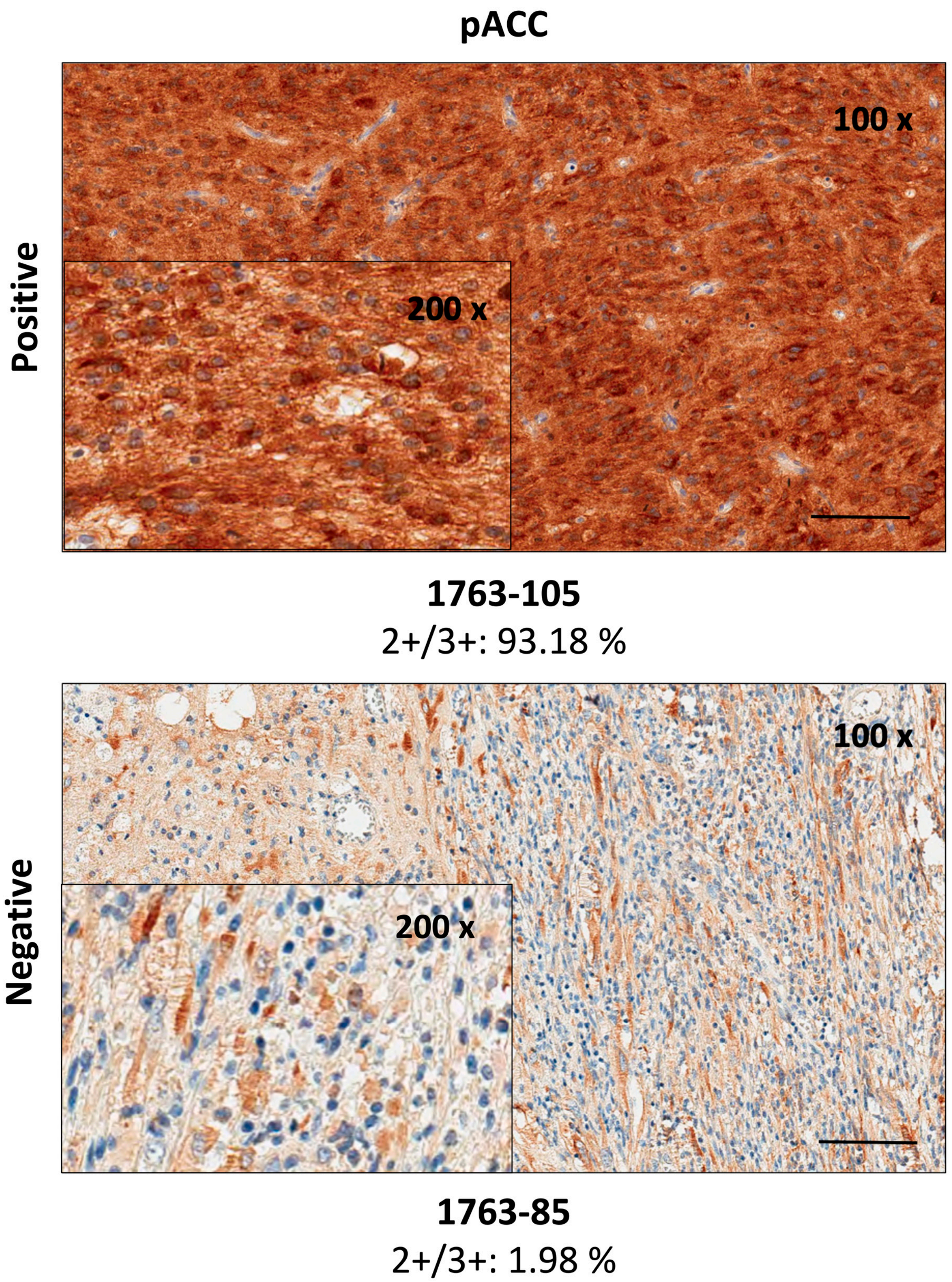

Supplementary Figure S2. Representative examples of pACC immunohistochemical staining in GBM tumor sections. High-magnification images (100× and 200×) illustrate two representative cases: a pACC-positive case (sample 1763-105, 2+/3+ positivity: 93.18%) and a pACC-negative case (sample 1763-85, 2+/3+ positivity: 1.92%). Cytoplasmic pACC immunoreactivity was quantified as the percentage of tumor cells with 2+ or 3+ staining intensity, using a dedicated cytoplasmic algorithm on ImageScope software (Leica Biosystems). A cut-off of 5% was applied to classify tumors as pACC-positive or pACC-negative, in accordance with previously published criteria. Scale bar represents 50 µm.

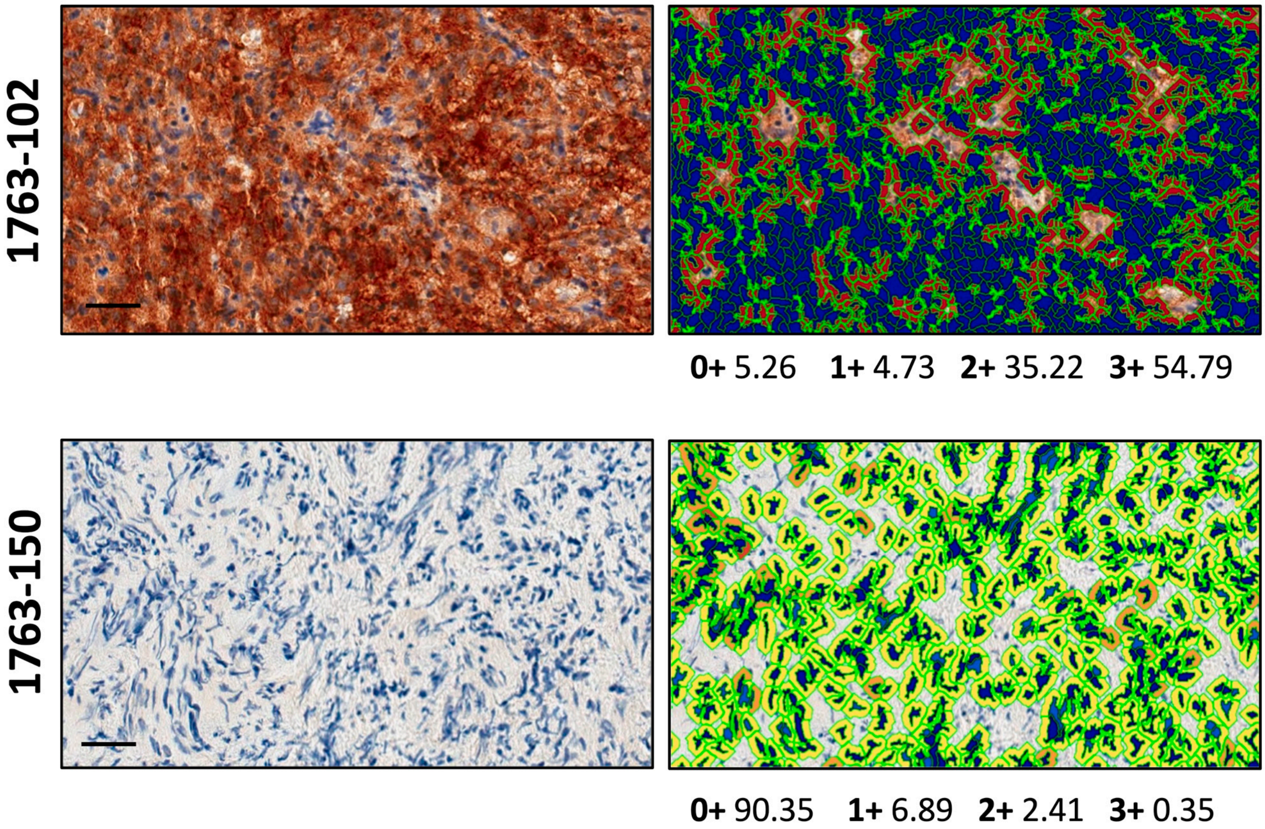

Supplementary Figure S3. Visualization of Aperio algorithms used to quantify pACC expression in tissue.
On the left representative unmasked sections (hematoxylin counterstain) and on the right the final mark-up of the images. Original magnification was 200x, scale bar represents 50 µm.

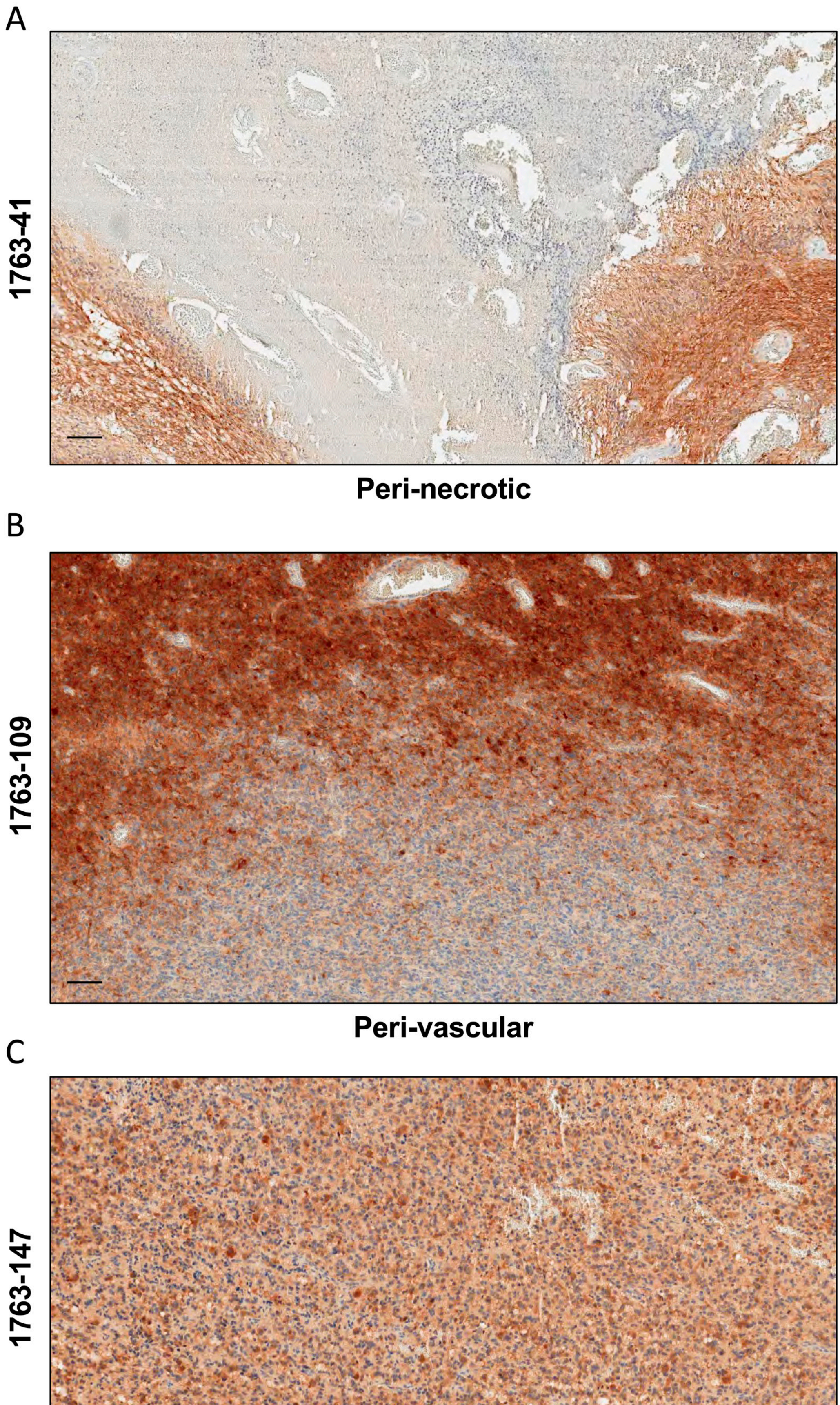
A
1763-41
Peri-necrotic
B
1763-109
Peri-vascular
C
1763-147
Homogeneous

Supplementary Figure S4. Representative pACC immunohistochemical staining patterns in GBM tumor sections. Heterogeneous spatial distribution of pACC expression was observed across tumor samples. Three distinct patterns are illustrated: **(A)** peri-necrotic pattern, characterized by reinforced cytoplasmic pACC immunoreactivity in tumor cells surrounding necrotic areas (sample 1763-41); **(B)** peri-vascular pattern, characterized by preferential pACC expression in tumor cells adjacent to vascular structures (sample 1763-109); **(C)** homogeneous pattern, characterized by diffuse and uniform cytoplasmic pACC positivity throughout the tumor parenchyma (sample 1763-147). All images were acquired at 50× magnification; scale bar represents 100 μm.

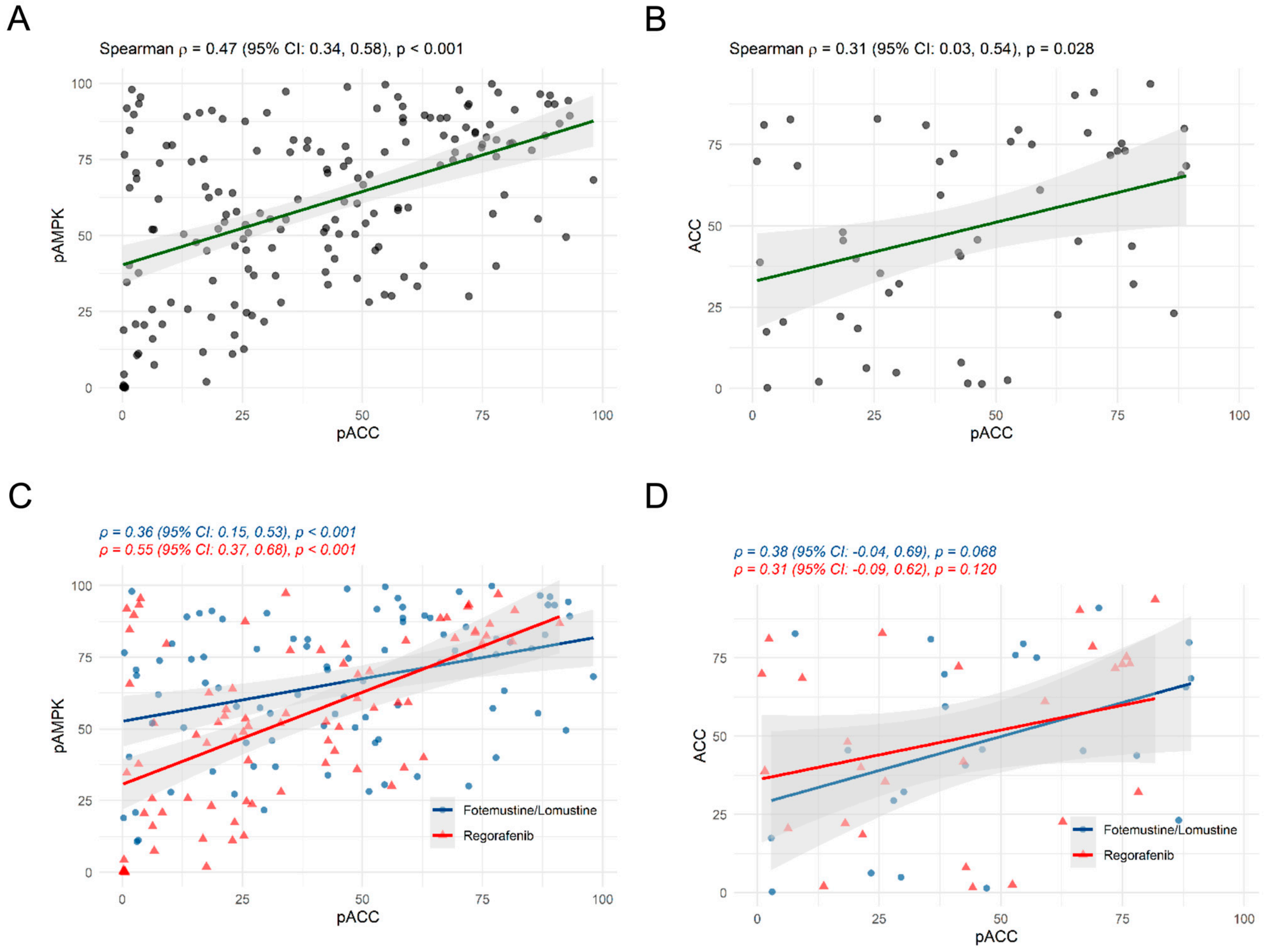

Supplementary Figure S5. Correlation between pACC and pAMPK expression across treatment groups. Scatter plots showing Spearman correlation between pACC (x-axis) and pAMPK or ACC (y-axis) immunohistochemical expression scores in glioblastoma specimens. **(A)** Significant positive correlation between pACC and pAMPK across the entire cohort ($\varrho = 0.47$, 95% CI: 0.34–0.58, $p < 0.001$). **(B)** Significant positive correlation between pACC and ACC in the overall cohort ($\varrho = 0.31$, 95% CI: 0.03–0.54, $p = 0.028$). **(C)** Stratified analysis by treatment group showing a significant positive correlation between pACC and pAMPK in both the fotemustine/lomustine group (blue; $\varrho = 0.36$, 95% CI: 0.15–0.53, $p < 0.001$) and the regorafenib group (red; $\varrho = 0.55$, 95% CI: 0.37–0.68, $p < 0.001$). **(D)** Stratified analysis showing a non-significant trend for the correlation between pACC and ACC in both the fotemustine/lomustine group (blue; $\varrho = 0.38$, 95% CI: −0.04–0.69, $p = 0.068$) and the regorafenib group (red; $\varrho = 0.31$, 95% CI: −0.09–0.62, $p = 0.120$). Solid lines and surrounding shaded areas represent linear regression fits with 95% confidence intervals overlaid solely for descriptive visualization of data trends and do not imply an inferential parametric linear model. Correlation coefficients and p-values were calculated using Spearman's rank correlation.

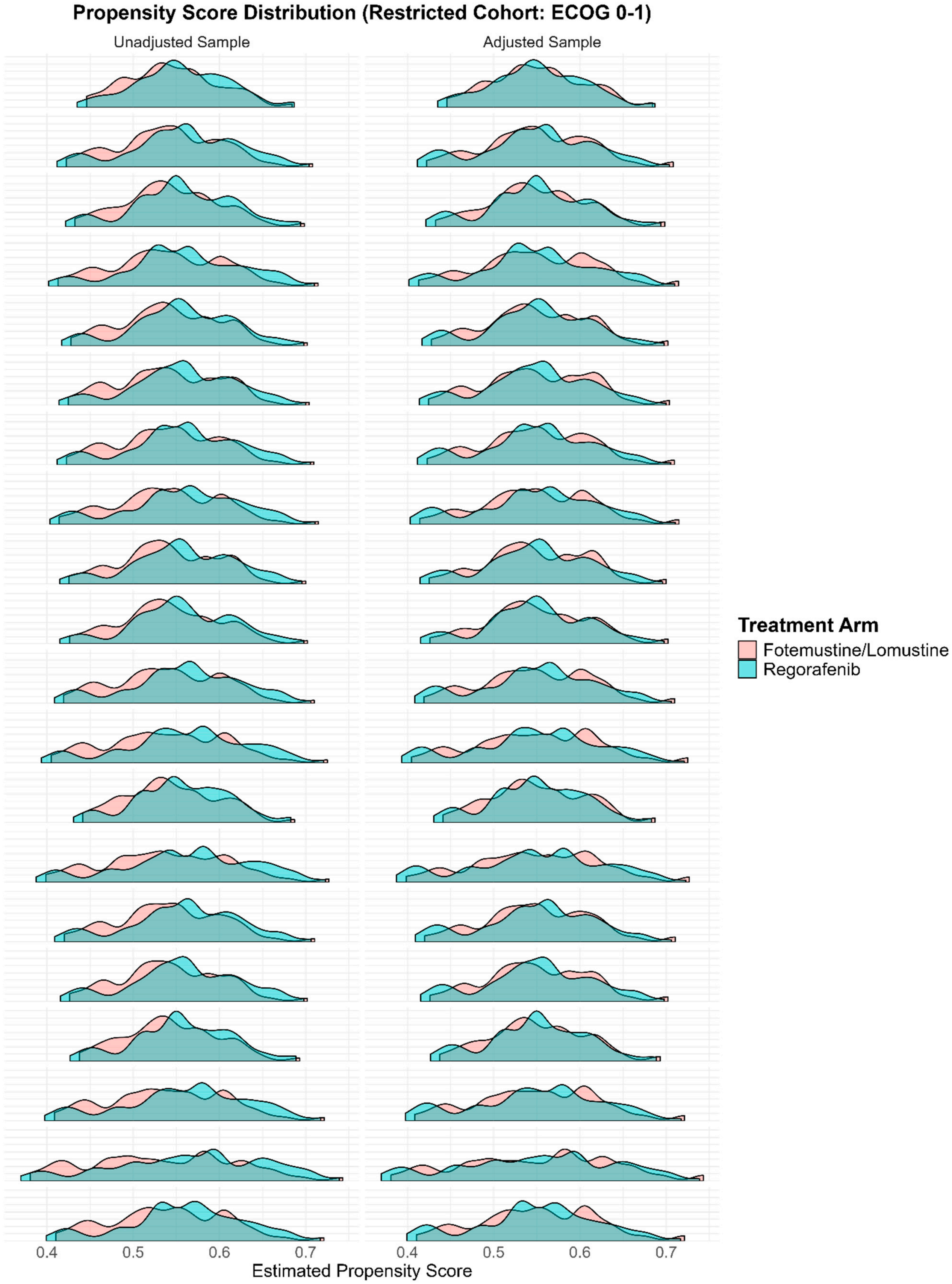

Supplementary Figure S6. Propensity Score Overlap (Common Support) Density Plot. Density distribution of estimated propensity scores for patients receiving regorafenib versus fotemustine/lomustine within the restricted cohort (ECOG PS 0–1, N = 136). The plot demonstrates strict overlap (common support) between the two treatment arms across the range of propensity scores, confirming the validity of the positivity assumption and ensuring that Inverse Probability of Treatment Weighting (IPTW) estimates do not rely on model-based extrapolation.

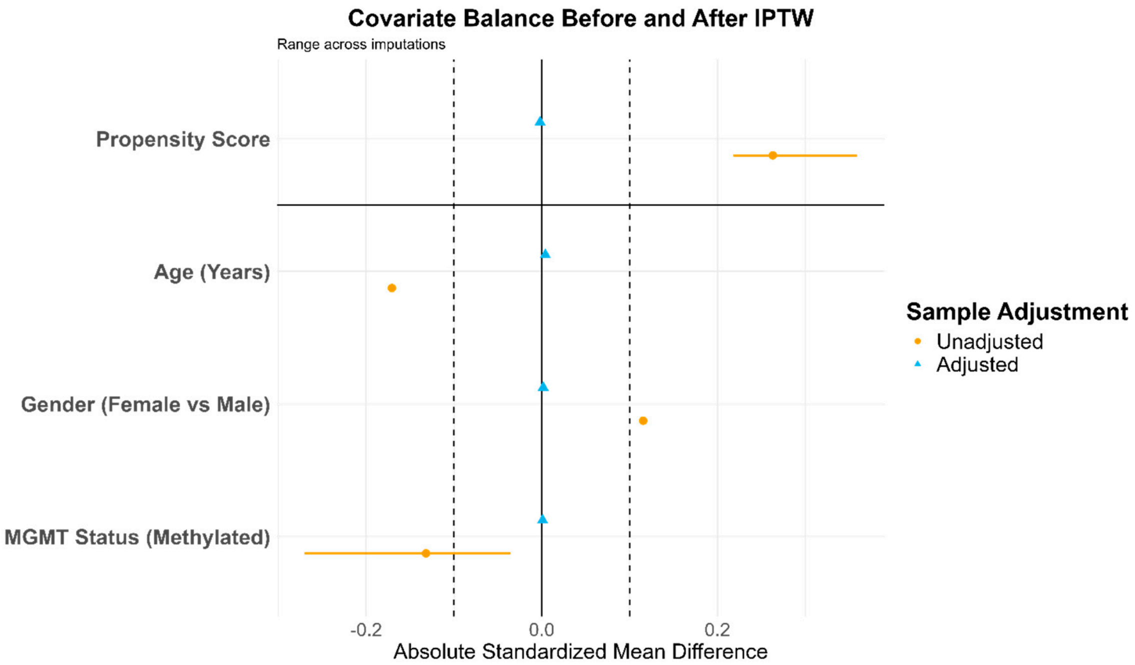

Supplementary Figure S7. Covariate Balance (Love Plot) Before and After IPTW. Standardized Mean Differences (SMD) for baseline covariates (age, gender, and MGMT methylation status) before (unadjusted) and after applying Inverse Probability of Treatment Weighting (IPTW) in the restricted cohort. The vertical dashed line represents the rigorous balance threshold of SMD = 0.1. After weighting, all covariates achieved optimal balance, effectively mitigating confounding by indication.

Subgroup	Regorafenib (Events/N)	Control (Events/N)	Unadjusted HR (95% CI)	Adjusted‡ HR (95% CI)	Unadj. p-int*	Adj.‡ p-int*
**pACC expression**					**0.605**	**0.587**
Negative (≤ 5.0%)	15/16	9/9	0.71 (0.31-1.63)	0.71 (0.29-1.77)		
Positive (> 5.0%)	66/68	81/81	0.90 (0.65-1.24)	0.92 (0.66-1.30)		
**pAMPK expression**					—	—
Negative (≤ 5.0%)	7/8	0/0	NE	NE		
Positive (> 5.0%)	74/76	90/90	0.89 (0.66-1.22)	0.91 (0.65-1.25)		

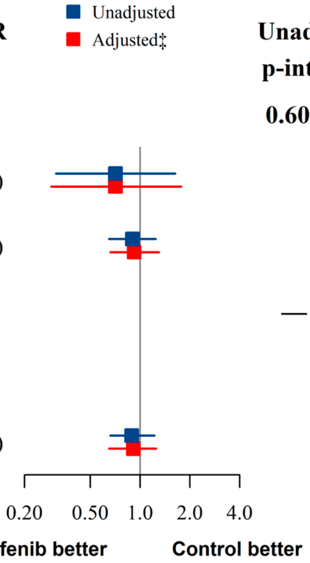

‡Adjusted for age, gender, ECOG PS, and MGMT methylation
**p-interaction calculated via likelihood ratio test (unadjusted) and Wald test (MICE pooled adjusted)*

Supplementary Figure S8. Subgroup analysis of progression free survival according to baseline pACC and pAMPK expression levels. Forest plot displaying unadjusted and adjusted hazard ratios (HR) for overall survival (OS) comparing second-line regorafenib with pooled control treatment (fotemustine/lomustine). For each subgroup, the number of events per total patients (Events/N), unadjusted HR (95% CI), multivariable-adjusted HR (95% CI), and interaction p-values are reported. CI, confidence interval; HR, hazard ratio; NE, not estimable. HRs < 1.0 indicate a survival benefit favoring regorafenib
